# Plant Surfaces: Structures and Functions for Biomimetic Innovations

**DOI:** 10.1007/s40820-016-0125-1

**Published:** 2017-01-04

**Authors:** Wilhelm Barthlott, Matthias Mail, Bharat Bhushan, Kerstin Koch

**Affiliations:** 1 Nees Institute for Biodiversity of Plants, Rheinische Friedrich-Wilhelms University of Bonn, Venusbergweg 22, 53115 Bonn, Germany; 2 Institute of Crop Science and Resource Conservation (INRES) – Horticultural Science, Rheinische Friedrich-Wilhelms University of Bonn, Auf dem Hügel 6, 53121 Bonn, Germany; 3 Nanoprobe Laboratory for Bio & Nanotechnology and Biomimetics, The Ohio State University, 201 W. 19th Avenue, Columbus, OH 43210-1142 USA; 4 Faculty of Life Sciences, Rhine-Waal University of Applied Sciences, Marie Curie-Straße 1, 47533 Kleve, Germany

**Keywords:** Bionics, Superhydrophobicity, Hierarchical structuring, Lotus effect, Salvinia effect, Evolution

## Abstract

An overview of plant surface structures and their evolution is presented. It combines surface chemistry and architecture with their functions and refers to possible biomimetic applications. Within some 3.5 billion years biological species evolved highly complex multifunctional surfaces for interacting with their environments: some 10 million living prototypes (i.e., estimated number of existing plants and animals) for engineers. The complexity of the hierarchical structures and their functionality in biological organisms surpasses all abiotic natural surfaces: even superhydrophobicity is restricted in nature to living organisms and was probably a key evolutionary step with the invasion of terrestrial habitats some 350–450 million years ago in plants and insects. Special attention should be paid to the fact that global environmental change implies a dramatic loss of species and with it the biological role models. Plants, the dominating group of organisms on our planet, are sessile organisms with large multifunctional surfaces and thus exhibit particular intriguing features. Superhydrophilicity and superhydrophobicity are focal points in this work. We estimate that superhydrophobic plant leaves (e.g., grasses) comprise in total an area of around 250 million km^2^, which is about 50% of the total surface of our planet. A survey of structures and functions based on own examinations of almost 20,000 species is provided, for further references we refer to Barthlott et al. (Philos. Trans. R. Soc. A 374: 20160191, 1). A basic difference exists between aquatic non-vascular and land-living vascular plants; the latter exhibit a particular intriguing surface chemistry and architecture. The diversity of features is described in detail according to their hierarchical structural order. The first underlying and essential feature is the polymer cuticle superimposed by epicuticular wax and the curvature of single cells up to complex multicellular structures. A descriptive terminology for this diversity is provided. Simplified, the functions of plant surface characteristics may be grouped into six categories: (1) mechanical properties, (2) influence on reflection and absorption of spectral radiation, (3) reduction of water loss or increase of water uptake, moisture harvesting, (4) adhesion and non-adhesion (lotus effect, insect trapping), (5) drag and turbulence increase, or (6) air retention under water for drag reduction or gas exchange (Salvinia effect). This list is far from complete. A short overview of the history of bionics and the impressive spectrum of existing and anticipated biomimetic applications are provided. The major challenge for engineers and materials scientists, the durability of the fragile nanocoatings, is also discussed.

## Introduction

**Fig. 1:**
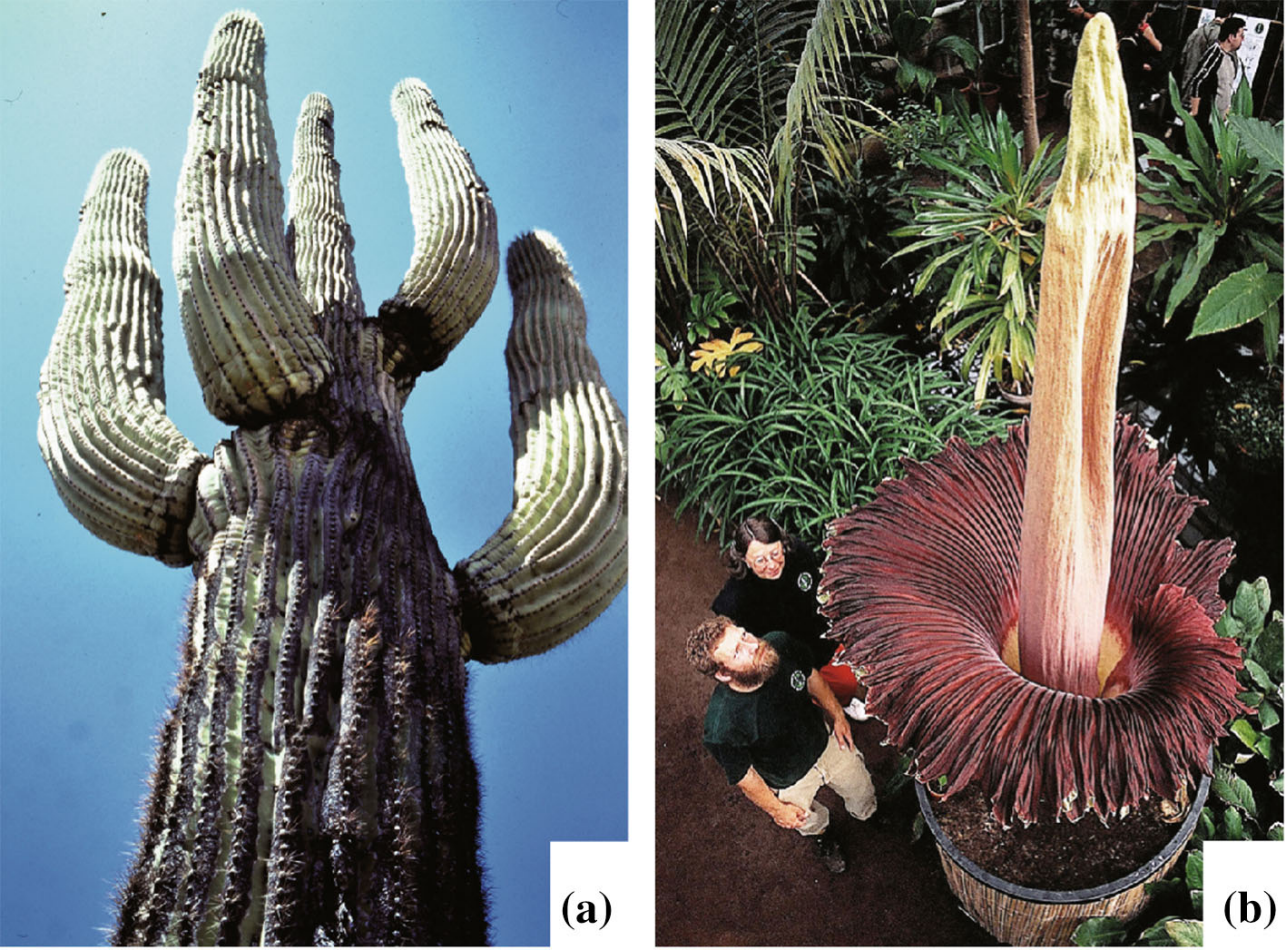
Hierarchical surface sculpturing of plants on the macroscopic scale. **a** The Saguaro (*Carnegiea gigantea*) is the largest cactus; it can grow up to 21 m tall. The stems are ribbed—even in the full sun of a desert in Arizona, large areas of the plant are *shaded*. At the same time, the ribs and elastic cuticle allow a rapid increase of the volume after sporadic rainfalls: the stems expand. Loss of water is a major problem for desert plants: the Saguaro is incrusted in a wax layer, but due to UV exposure the surfaces age and become wettable. Saguaros can live 150 years or more. **b** In contrast, the Giant Arum (*Amorphophallus titanum*) lives in the deepest shadows of the humid rain forest understories in Sumatra. Its flower opens for only one to two days; it reaches a height of three meter and is the largest blossom in the plant kingdom. The giant pleated “petal” (spathe) weights less than one kg: the largest light-weight construction amongst plants, possibly even in any organism. The riblets serve as mechanical stabilizers: when the spathe opens, its surface is hydrophobic to shed rain droplets. Very unusual wax crystals occur on the unpleasant smelling central column, which heats periodically to almost 40 °C to generate a convection flow to attract insects

Surfaces define the boundaries for the well-structured world of solids, and it is surfaces that define their interactions. They play crucial roles in environmental interactions. This is of particular importance for sessile organisms with large functional surfaces: plants. Green plants cover the terrestrial biomes of our planet and—not surprisingly—show a stunning diversity of hierarchical surface structures which has been revealed with the help of scanning electron microscopy techniques (SEM) first employed in the 1970s (survey in Ref. [2]). It is even possible to examine the hierarchical surface structures at the macroscopic scale, as illustrated in two of the giants in the plant kingdom; the Saguaro cactus (Fig. 1a) and the Titan Arum (Fig. 1b). On the other hand, the details of structures like wax crystals on their surface (Figs. 4h and 7) are only revealed by scanning electron microscopes.

**Fig. 2:**
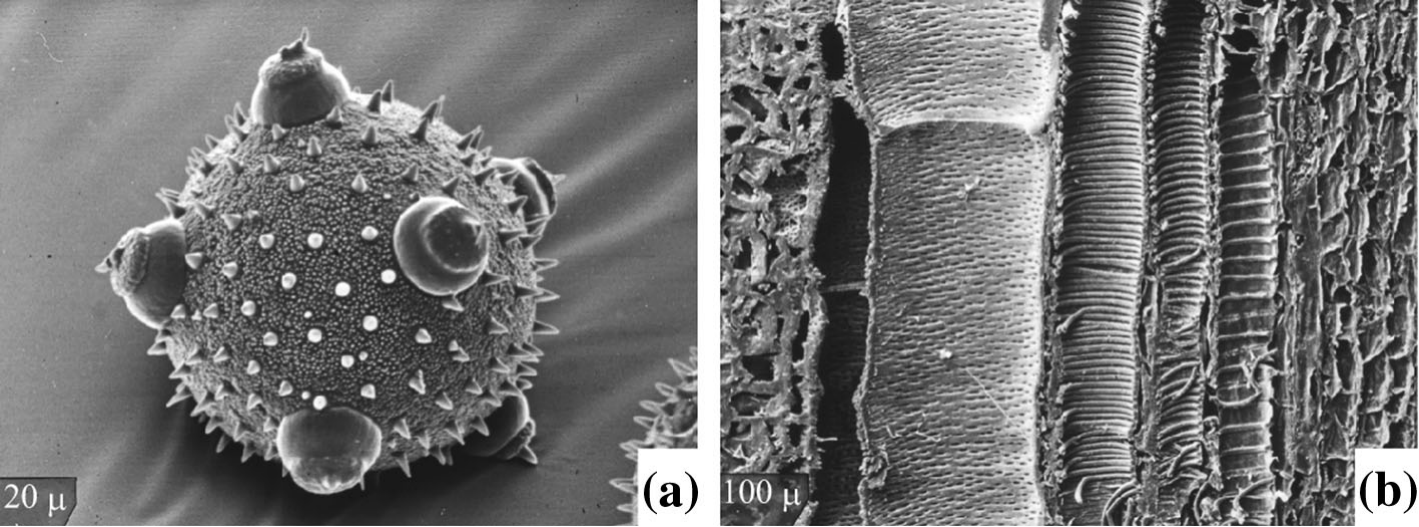
Internal (“non-cuticular”) functional surfaces are usually hydrophilic, two examples from a squash or pumpkin (*Cucurbita pepo*) are illustrated: **a** pollen grain, its surface functions are connected with attachment and detachment to the pollinating insect and the stigma of the pumpkin flower, and possibly temperature control under insolation. In wind-dispersed pollen, these structures might also increase the Reynolds-numbers, **b** a vessel-element of the same plant exhibiting complex spiral and perforated structures to transport water within the plant. The structural elements of internal surfaces fundamentally differ from the outer cuticular surfaces (compare, e.g., Figs. 3, 7, 14, and 15)

**Fig. 3:**
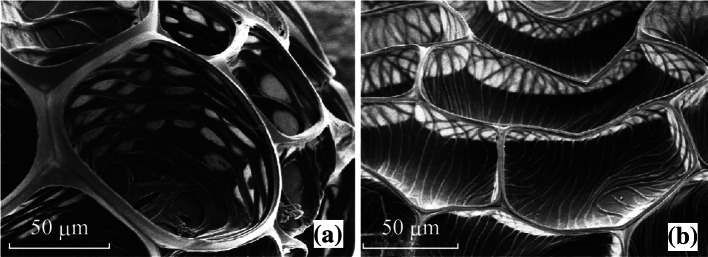
SEM micrographs of two seed surfaces with concave cell sculpturing. The miniature seeds of both species: **a** *indica* and **b** *Triphora trianthophora* are optimized for seed dispersal by wind. They are hydrophobic and float for a short time in water (compare *Aeginetia* in Fig. 27): the concave sculpture of the non-living cells can be interpreted as a shrinkage deformation during seed maturing and drying. The bands which form an inner network in (**a**), and the surface pattern in (**b**) are built by cellulose. All these features are light-weight constructions and generate high Reynolds-numbers to prolong the floating time

Pollen and spores exhibit particularly refined hierarchical structures; they are distinctive from all other plant surfaces like leaves. The functional properties of pollen (e.g., the pollen of Cucurbita pepo, Fig. 2a) are associated with the attachment and detachment to the pollinating insect and the stigma of the flower, and possibly temperature control under insolation. Pollen are hydrophilic, spores are occasionally superhydrophobic, even bacterial spores. Wind-dispersed miniature “dust seeds” (e.g., the seeds of *Aeginetia* and *Triphora,* Fig. 3) are dispersed by air and possess a surface roughness to increase their Reynolds number for long distance dispersal. Pollen and spores are not discussed in this chapter: palynology is a very specialized field and a vast literature exists (e.g., Ref. [3, 4]). Internal “non-cuticular” surfaces (Fig. 2) like pollen or conducting vessels are also not considered in the following chapters, they are multifunctional and different form “outer” cuticular surfaces of plants. Strangely enough biomimetics has little interest in this particular subject, although applications are plausible.

**Fig. 4:**
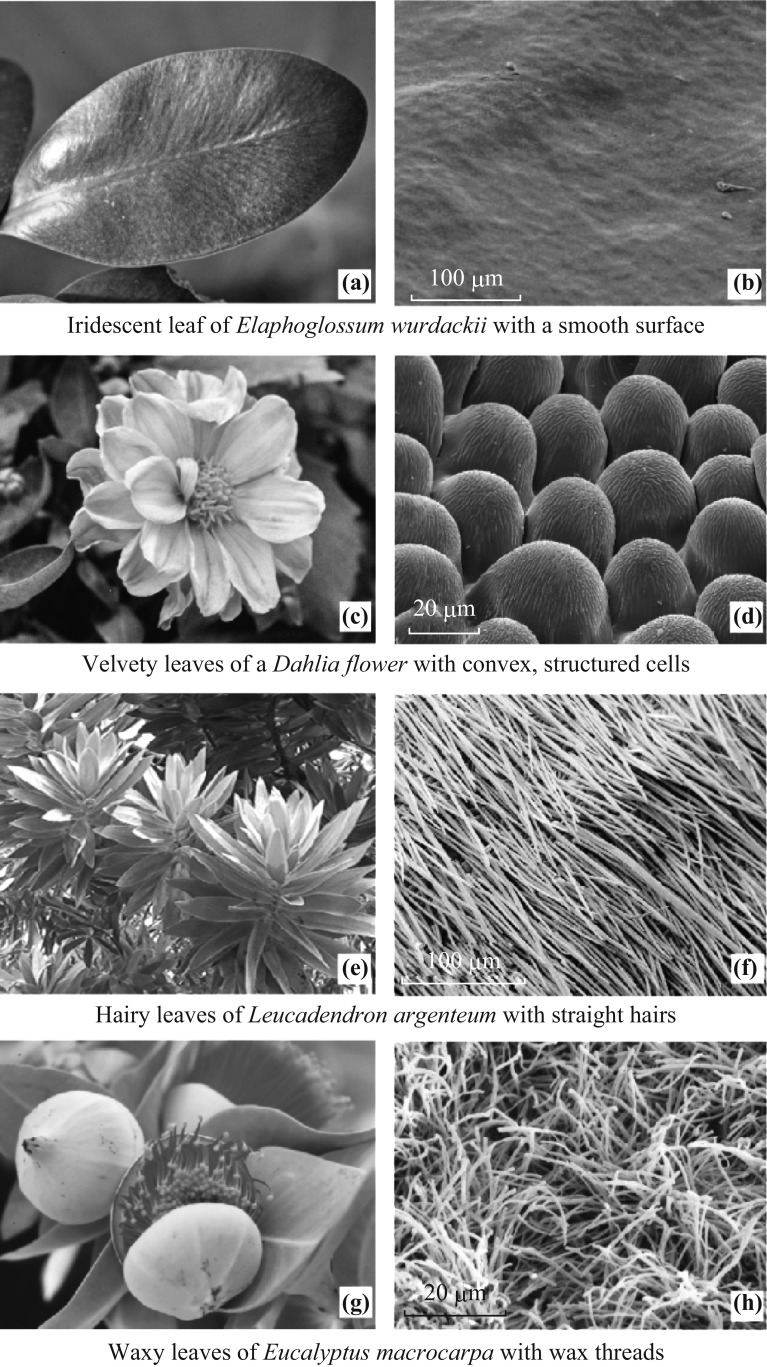
Macroscopic optical appearance of plant surfaces and their surface micro-structures. (**a**) The leaves of *Elaphoglossum wurdackii* appear glossy because of a flat surface structure shown in (**b**), their iridescence is caused by thin layers within the cuticle. In (**c**) the flower petals of *Dahlia* appear velvety due to the convex microstructure of the epidermis cells, shown in (**d**). In **e** the silvery appearance of the *Leucadendron argenteum* leaves is caused by a dense layer of light reflecting hairs (**f**). In (**g**) the leaf and flower bud surfaces of *Eucalyptus macrocarpa* appear white or bluish, caused by a dense covering with thread-like wax crystals (**h**)

**Fig. 5:**
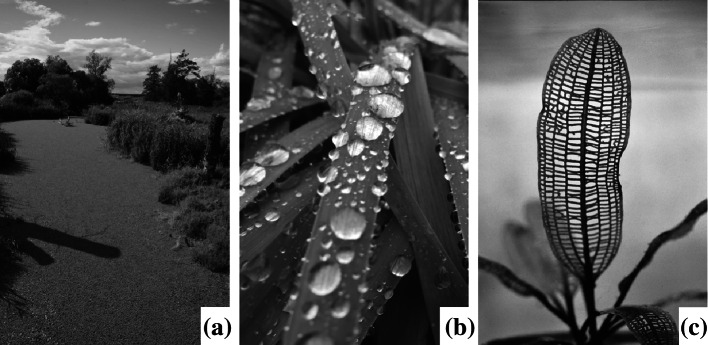
Plant surfaces exposed to the air are very often superhydrophobic. Like the leaves (**a**) of the reed (*Phragmites australis*) and the grassland in the background, or the surfaces of the floating fern (*Salvinia natans*) in the foreground left in the Oder national park, Germany. Dew droplets in the early morning roll-off of the superhydrophobic grass leaves (**b**). Since grasslands alone forms the largest terrestrial ecosystems (ca. 52.5 million km^2^) of our planet, we estimate there are at least about 250 million km^2^ of superhydrophobic leaf surfaces, which equal about 50% of the earth's total surface. But all plant roots are superhydrophilic like the surfaces of water plants, illustrated in (**c**): The Madagascar Laceleaf (*Aponogeton madagascariensis*) additionally exhibits a grid of a lattice-like network to reduce the flow resistance. *Source* **a** kindly provided by Pierre Ibisch

The diversity of plant surface structures arises from the variability of cell shapes, and hierarchically superimposed micro- and nanostructures of the cell surfaces (mainly wax crystals), and by the formation of multicellular structures (Fig. 4) [2, 5, 6]. Based on these cellular and sub-cellular units a nearly unlimited combination of structures leads to a high structural and functional diversity of the surfaces of the some 450,000 different species of land plants [1]. Superhydrophobicity is one of the most remarkable features of many plant surfaces; most families of higher plants [1] include many species where the entirety or part (e.g., only lower side of the leaves) of the assimilating leaf surface is superhydrophobic. Grasses (Fig. 5a, b) with a few exceptions (e.g., Maize), are superhydrophobic and are with around 12,000 species, one of the largest plant families which dominates the largest ecosystems of our globe. As is the case in many plants, often only the young leaves are superhydrophobic (Fig. 5b), older ones may become wettable: superhydrophobicity is often an instable state in plant surfaces (compare also the Saguaro, Fig. 1a)—as is also seen in technical surfaces. Some grasses (like *Elymus arenarius* with a static contact angle of 161°) exhibit similar properties to lotus leaves [7]. One square meter of a meadow may exhibit a minimum of four square meters of leaf surfaces (compare, e.g., Ref. [8]). According to FAO assessments [9], grasslands (e.g., steps, savannahs, wheat, and rice fields) cover some 52.5 million square kilometers. A rough calculation indicates that at least 250 million square kilometers (with other plant families included, possibly much more) are superhydrophobic: this means more than half of the total surface area of our whole planet. In all actuality, the dimensions are probably considerably higher and might equal the total surface of our planet.

**Fig. 6:**
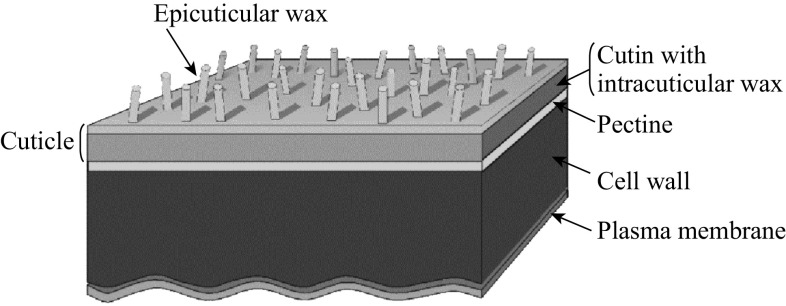
A simplified model of the stratification of the outermost layers of a plant epidermal cell. The schematics shows the outermost wax layer in its most common form, as composite of three-dimensional waxes with an underlying wax film. Below this layer is the cuticula, made of a cutin network and integrated waxes. The cuticula is connected with the underlying cellulose wall by a pectin layer. Below the cell wall, the plasma membrane is shown. This membrane separates the water-containing living part of the cells from the outermost non-living outer cell wall and cuticle, as shown in the schematic

There is a basic and obvious difference in surface structure and function between aquatic and terrestrial plants. In terrestrial (vascular) plants an epidermis is present, as the specialized outermost cell layer with a cuticle of the primary tissues of all leaves and several other organs it plays an important role in environmental interactions and surface structuring. A simplified model presented in Fig. 6 shows a layered stratification of the outermost part of epidermis cells. Starting with the outside, one finds a highly functional thin outermost layer, the polymer cuticle with its superimposed waxes. This outermost layer covers nearly all aerial tissues of land-living plants as a continuous extracellular membrane, but is absent in roots. One of the most important attributes of the cuticle is its function as a transpiration barrier, which enables plants to overcome the physical and physiological problems connected to an ambient environment, such as desiccation.

The cuticle is basically a biopolymer made of polyester called cutin, impregnated with integrated (intracuticular) waxes. Additionally, waxes on the cuticle surface (epicuticular waxes) play an important role in surface structuring at the sub-cellular scale. They occur in different morphologies, show a large variability in their chemistry, and are able to self-assemble into three-dimensional crystals. Intracuticular waxes function as the main transport barrier to reduce the loss of water and small molecules such as ions from inside of the cell, and also for reducing the uptake of liquids and molecules from the outside. Epicuticular waxes form the boundary layer for many interactions with the plant´s environment, like wettability or spectral reflection (see Sect. 6). The next layer (Fig. 6) is the pectin layer. It connects the cuticle to the much thicker underlying cellulose wall, which is built by single cellulose fibrils. Pectin is not always formed as a layer, but in some species, especially during the early ontogeny of the cuticle, a layered structure has been shown by transmission electron microscopy (TEM). Additionally polysaccharides, not shown in this schematic, are integrated into the cellulose wall. The last layer shown is the plasma membrane, which separates the living, water-containing compartment cell from the outer non-living part of the epidermis.

We focus on *superhydrophobic* and *superhydrophilic* surfaces, which are of particular importance for biomimetic applications (e.g., self-cleaning: lotus effect). *Superhydrophilicity* means, a droplet imposed on a surface “spreads” instantly and a contact angle cannot even be measured, e.g., in the leaves of *Ruellia* [10]. In contrast, on a *superhydrophobic* surface water remains as an almost globular droplet with a contact angle of more than 150°. The SEM micrographs presented were largely taken from our archive of almost 220,000 SEM micrographs at the University of Bonn which has been built up as a result of over four decades of research on biological surfaces (compare Ref. [1]) by the first author and his collaborators.

Biomimetics and bionics (which we consider here as synonymous) are surmised to be modern scientific fields; despite the evidence that inspiration from living organisms is as old as mankind. The magnificent 17,000-year-old paleolithic paintings in the caves of Lascaux are bioinspired—like the Cadillac tail fins in the 1960s. Bio-inspiration in the sense of non-functional “biodecoration” is an inspiration for art and design into modern times [11, 12]. Early attempts to copy mechanical functions were not particularly successful—Ovid’s story of Daedalus and Icarus and Leonardo da Vinci´s design of flying machines and other devices did not translate into technical success stories.

Historically, the dream of flying and the use of the strange phenomena “electricity” were the two fundamental forces for the foundation of what we call today *bionics* or *biomimetics.* The construction of an electric battery based on observations of the *Torpedo* fish (today we call it Electric Ray) by Alessandro Volta in 1800 was the first milestone [11] of bionics. And Icarus´ dream was realized with the first well-documented, repeated, and successful flights by Otto Lilienthal from 1894 onwards; his design was based on his analysis of the flight of birds. The term “Biotechnik” (usually abbreviated in German as “Bionik”) for the new field was coined by Raul Francé in 1920 [13] and finally rediscovered under the influence of cybernetics under the name “Bionics” [14] and “Biomimetics” between 1960 and 1964; the misleading term “Biomimicry” arose in 1982 (for a historical survey see Ref. [11]). Surfaces came surprisingly late into the focus of bionics: The Swiss engineer George de Mestral observed in 1941 the way that the burrs (*Arctium*) clung to his trousers and his dog—in 1958, he developed the bionic *hook-and-loop* fastener under the trade mark Velcro^®^. Starting with the discovery of hierarchically structured superhydrophobic lotus-surfaces [2, 15, 16] and the drag-reducing shark skin [17, 18], biomimetic surface technologies (e.g., lotus-, shark-, gecko-, moth eye-, and salvinia-effect) became a most important field [1, 19, 20]. The publication of the “Lotus Effect^®^” in 1997 [15] led to a change of paradigms in surface technologies [1]. Biological role models provide an extraordinary diversity for innovative surface technologies, which are described for plants in the following chapters primarily under the view of biologists.

This paper is completely based on our Sect. 3.6 “Plant Surfaces: Structures and Functions for Biomimetic Applications” in the 4th edition of B. Bhushan, Handbook of Nanotechnology (Springer 2017) [21].

## Chemistry of Plant Surfaces

**Fig. 7:**
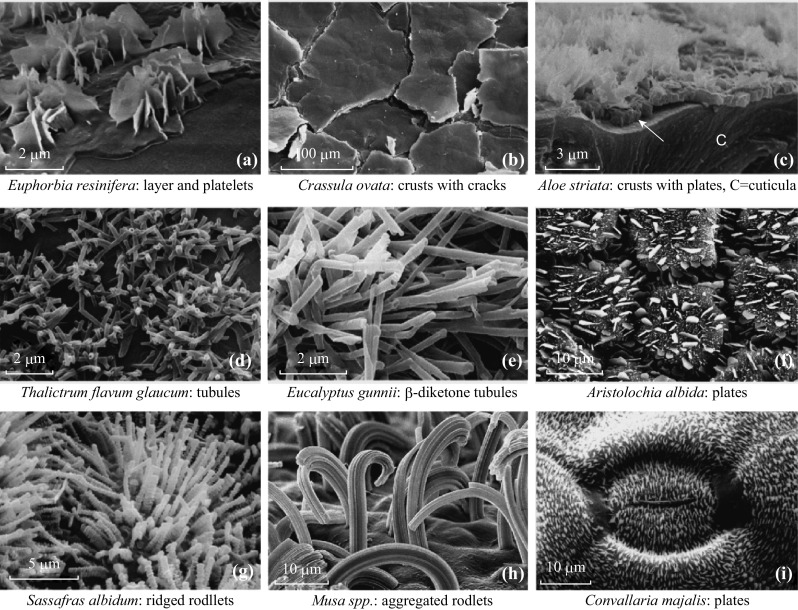
SEM micrographs of epicuticular waxes: in (**a**) waxes on a leaf of *Euphorbia resinifera* have been particularly removed to show the composite structure of a basal wax layer with three-dimensional wax platelets on it. In (**b**) a wax crust with fissures on a leaf of *Crassula ovata* is shown. A cross section through the periclinal wall of *Aloe striata* (**c**) shows the cuticle (indicated by C) and a wax layer (indicated by an arrow) with wax platelets on top. In (**d**) nonacosanol tubules on *Thalictrum flavum glaucum* leaves and (**e**) ß-diketone wax tubules on *Eucalyptus gunnii* leaves are shown. In (**f**) wax platelets on *Aristolochia albida* leaf and in (**g**) transversely ridged rodlets on a leaf of *Sassafras albidum* are shown. In (**h**) longitudinally aggregated wax threads form large aggregated rodlets on the lower side of the leaves of *Musa* species (spp.). In *Convallaria majalis* leaves, shown in (**i**), wax platelets are arranged in a pattern, similar to magnetic field lines, around the stomata. Thin wax films are not visible in SEM, but are present below and between the three-dimensional waxes shown here

Here, we consider only the surfaces of higher or vascular plants (Tracheophyta). Primarily aquatic plants (from unicellular algae to seaweeds, see Sect. 8) lack a cuticle and have very differing superhydrophilic surfaces. For biomimetic applications, vascular plants are most important. In land or vascular plants, waxes from monomolecular layers to thick crusts or 3D-crystals, form the boundary layer of the surface (Fig. 7). They are sometimes visible as a white or bluish coloration of leaves and fruits, as in wheat or cabbage, grapes, or plums. These colorations are caused by reflection of parts of the visible light spectrum by a dense coverage of three-dimensional (3D) wax structures. The fan palm *Copernicia prunifera,* the natural source of carnauba wax, has massive crusts of epicuticular wax, weighing several mg cm^−2^. Carnauba wax is commercially used, e.g., for car and furniture polishes, medical products, and candy. Even when there is not a bluish coloration visible, three-dimensional waxes are often present. In plants, three-dimensional waxes are responsible for several surface functions. Waxes are not only an essential part of the plant cuticle, but can also be found in fungi, lichens, and animals. Waxes occur as filling material within the basic cutin network (intracuticular), and are also found on top of the cuticle (epicuticular). The epicuticular waxes occur in very differing morphologies, all of which are crystalline and thus self-assembling (Fig. 7) (survey in Ref. [22]). However, waxes of different plants, and also waxes of different parts of a plant, vary in their morphology and chemical composition—they are absent in roots. In general, plant waxes are mixtures of long-chain hydrocarbons and their derivatives, and in some species they also contain cyclic compounds. Because of the strong correlation between the wax crystal morphology and their chemical composition, some waxes, such as the nonacosan-10-ol tubules of the Lotus leaves and many other plants, have been named after their main wax constitution [23].

### Chemical Composition of Wax

The term “wax” is used for a variety of biogenic products that contain fatty materials of various kinds. Well-known examples are bees wax, paraffin, and carnauba wax from wax palms (*Copernicia prunifera*). Plant waxes are mixtures of aliphatic hydrocarbons and their derivatives, with carbon chain lengths between 20 and 40, and in the case of esters (two connected chains) about 60 atoms. Several reviews have addressed the chemical composition of plant waxes [24–26]. The chemical composition of plant waxes is highly variable amongst different plant species, or within the organs of one species (e.g., upper or lower side of leaves) and even during organ development [27]. The main component classes are primary and secondary alcohols, ketones, fatty acids, and aldehydes. Alkanes are very common in plant waxes, but usually occur in low concentrations. Other compounds are more rarely found in plant waxes, but in those waxes where they occur, they may be the dominant compound. The most common wax compounds and their typical chain length are shown in Table 1. Examples of commonly found waxes and their major compounds are presented in Table 2. For some of those waxes, it has been shown that their dominant compounds crystallize in the same morphology as the complete wax mixture. Examples are the primary alcohols and the β-diketone waxes found on different parts of wheat plants [28]. However, an increasing number of publications report the discovery of new wax components and a long list of rare and uncommon ingredients, such as methyl-branched aliphatics [29]. Environmental factors, such as temperature or light intensity, influence the quantity of waxes and their chemical composition [30–32].

**Table 1:** The most common chemical compounds in plant waxes and their spectrum of chain length

		Chain length
1 Aliphatic compounds		
1.1 In waxes frequently existing, but mostly as minor compounds		
Alkanes	CH_3_–(CH_2_)n–CH_3_	Odd C_19_–C_37_
Primary alcohols^a^	CH_3_–(CH_2_)n–CH_2_–OH	Even C_12_–C_36_
Esters	CH_3_–(CH_2_)n–C0–0–(CH_2_)m–CH_3_	Even C_30_–C_60_
Fatty acids	CH_3_–(CH_2_)n–COOH	Even C_12_–C_36_
Aldehydes	CH_3_–(CH_2_)n–CHO	Even C_14_––C_34_
1.2 In waxes rarely existing, but if, than as major wax compounds		
Ketones e.g., palmitones	CH_3_–(CH_2_)n–CO–(CH_2_)m–CH_3_	Odd C_25_–C_33_
ß–diketones	CH_3_–(CH_2_)n–CO–CH_2_–CO–(CH_2_)m–CH_3_	Odd C_27_–C_35_
Sec. alcohols e.g., nonacosan-10-ol	CH_3_–(CH_2_)n–CH_2_OH–(CH_2_)m–CH_3_	Odd C_21_–C_33_
2 Cyclic Compounds		
Flavonoids	e.g., Quercetin	
Triterpene	e.g., ß-Amyrin	

^a^Primary alcohols are common minor constitutions in waxes, but can occur as major compounds in the wax, e.g., of grasses, eucalypts, clover, and other legumes [26]. Further examples of occurrence are given in Table 2

**Table 2:** Common wax types in plant species and their major chemical compounds

Wax type	Species	Dominating chemical compound(s)
Films	*Hedera helix*	Prim. alcohols, aldehydes
Films	*Magnolia grandiflora*	Fatty acids C_24_–C_30_, prim. alcohols C_24_–C_28_
Films	*Prunus laurocerasus*	Alkanes C_29_, C_31_
Crust	*Crassula ovata*	Aldehydes C_30_, C_32_, alkane C_31_
Diketone tubules	*Eucalyptus globulus*	Beta-diketones C_33_
Diketone tubules	*Leymus arenarius*	Beta-diketone C_31_, hydroxy-beta-diketone C_31_
Nonacosanol tubules	*Ginkgo biloba*	Sec. alcohol C_29_
Nonacosanol tubules	*Nelumbo nucifera*	Sec. alkanediols C_29_
Nonacosanol tubules	*Thalictrum flavum glaucum*	Sec. alcohol C_29_
Nonacosanol tubules	*Tropaeolum majus*	Sec. alcohol C_29_
Nonacosanol tubules	*Tulipa gesneriana*	Sec. alcohol C_29_
Platelets	*Convallaria majalis*	Prim. alcohol C_26_, C_28_, aldehydes
Platelets	*Euphorbia myrsinites*	Prim. alcohol C_26_, aldehydes
Platelets	*Galanthus nivalis*	Prim. alcohol C_26_
Platelets	*Iris germanica*	Prim. alcohol C_26_
Platelets	*Triticum aestivum*	Prim. alcohol C_28_
Transversely ridged rodlets	*Aristolochia tomentosa*	Ketones
Transversely ridged rodlets	*Gypsophila acutifolia*	Alkanes C_31_
Transversely ridged rodlets	*Liriodendron chinense*	Ketones
Longitudinal ridged rodlets	*Benincasa hispida*	Triterpenol acetates

With exception of the fruit surface of *Benincasa hispida*, data represent the waxes on the leaves of the species. All references for the chemical data are listed in [42] and examples of the wax types here listed are shown in Fig. 7

Many or even most plant “waxes” do not match the chemical definition of true waxes and they are usually complex mixtures of differing compounds. For example, triterpenoids are cyclic hydrocarbons, which occur in high concentrations in the epicuticular coatings of grapes (*Vitis vinifera*) [30]. Other plant waxes contain polymeric components such as polymerized aldehydes which are only slightly soluble in chloroform [33, 34]. It should be noted that nearly all the existing data of the chemical composition of plant waxes are based on solvent-extracted waxes. These are mixtures of epicuticular and intracuticular waxes, which may be chemically different, as shown for the waxes of *Prunus laurocerasus* by Jetter and Schäffer [27] and by Wen et al. [35], for *Taxus baccata*. The development of more selective methods of wax sampling allows selective removal of the epicuticular waxes and their analysis separately from the intracuticular wax fractions [27, 36].

Epicuticular wax structures usually occur in the size ranging from 0.2 to 100 µm (Fig. 7); thus, the appropriate microscopic techniques for investigation of their morphology are SEM and low pressure- or environmental SEM. Several SEM investigations showed that most of the epicuticular waxes form three-dimensional structures, with great variations of their morphologies. Comprehensive overviews of the terminology and micromorphology of epicuticular waxes are given by Barthlott et al. [22], Jeffree [26], and in Ref. [37]. The comprehensive classification of Barthlott et al. [22], which we follow here, includes 23 different wax types. It is based on chemical and morphological features and also considers orientation of single crystals on the surface and the orientation of the waxes to each other (pattern formation). In this classification, the wax morphologies include thin films and several three-dimensional structures such as crusts, platelets, filaments, rods, and tubules which have a hollow center. Morphological sub-types are, for example, entire and non-entire wax platelets. A further sub-classification is based on the arrangement of the crystals, e.g., whether they are randomly distributed, in clusters, in parallel orientation, or in specific arrangements around stomata, as the “Convallaria” type (Fig. 7h). The most common wax morphologies are introduced in the following section and are shown in Table 2.

**Fig. 8:**
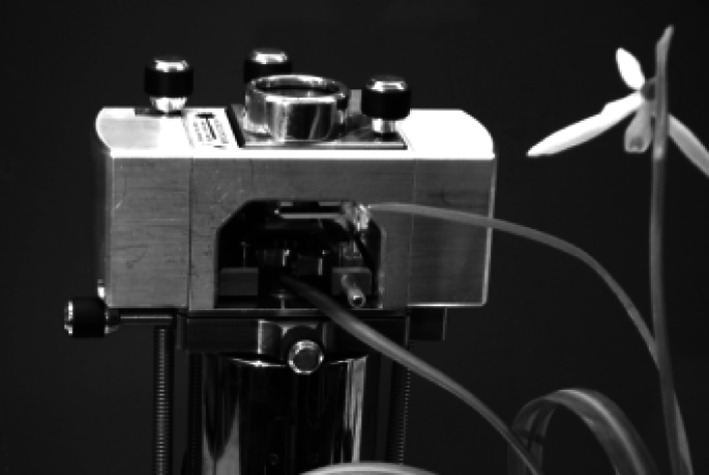
FM experimental set-up for long-term investigations of wax crystallization on a living plant surface. The tip of the leaf of a snowdrop (*Galanthus nivalis*) has been fixed on the specimen holder with a drop of two-compound glue. Existing waxes have been removed, and the rebuilding (self-healing) of the wax was studied over several hours. Appropriate scan conditions for living plant surfaces are given in the text, and the method of wax removal is described in detail in [40]

Probably all terrestrial plant surfaces are covered by thin (in the extreme monomolecular) wax films, the three-dimensional wax crystals appear on underlying wax film as shown in Fig. 7a for the waxes of *Euphorbia resinifera* and has been reported for several species [22, 38–41]. Wax films are often incorrectly referred to as “amorphous” [42]. On several plant surfaces, wax films are limited to a few molecular layers which are hardly visible in the SEM. By mechanical isolation of the epicuticular 3D waxes, e.g., freezing in glycerol, the waxes can be removed from the cuticle, and transferred onto a smooth artificial substrate for microscopic investigations [36]. With this method, the remnants of the wax film can be detected, and the film thicknesses can be determined. Wax film formation has been investigated on a living plant surface by atomic force microscopy (AFM, shown in Fig. 8) [40, 43]. Such investigations show that wax films are composed of several monomolecular layers, with thicknesses up to several hundred nanometers. In the following, these relatively thin wax films (<0.5 µm) are called two-dimensional (2D) waxes, and the thicker wax layers (0.5–1 µm) and wax crusts (>1 µm) are called three-dimensional (3D) waxes. Wax crusts are often found in succulent plants, as on the leaves of *Crassula ovata*, shown in Fig. 7b. Such a multilayered assembly of waxes is detectable by a cross section through the epidermis, as shown in Fig. 7c for *Aloe striata.*

Three-dimensional waxes occur in different morphologies. Most common are tubules, platelets, rodlets, and longitudinally aggregated rodlets shown in Fig. 7d–i.

Wax tubules are hollow structures, which can be distinguished chemically and morphologically. The first type, called nonacosanol tubules, contains large amounts of asymmetrical secondary alcohols, predominantly nonacosan-10-ol and its homologues and to a certain degree also asymmetrical diols [23, 44, 45]. Nonacosan-10-ol is the most common “waxy” coating of all major vascular plant groups and was evolved with the conquest of land some 450 million years ago, a phylogenetic tree is provided by Ref. [1]. The nonacosanol tubules are usually 0.3–1.1 µm long and 0.1–0.2 µm wide. The second type of tubules contains high amounts of *ß*-diketones, such as hentriacontane-14,16-dione [46]. This particular kind of wax tubule is characteristic for many grasses (Poaceae) and also occurs in various other groups [47]. Figure 7e shows that the *ß*-diketone tubules are two to five times longer than the nonacosanol tubules shown in Fig. 7d. Their length reaches from 2 to 5 µm, and diameters vary between 0.2 and 0.3 µm.

Platelets, as shown in Fig. 7f, are the very common wax structures found in all major groups of plants. Following the terminology of Barthlott et al. [22], waxes are termed platelets when flat crystals are connected with their narrow side to the surface. Platelets can be further differentiated by their outline into, e.g., entire or undulated ones. Platelets vary considerably in shape, chemical composition, and spatial pattern. For platelets, only limited information about the connection between morphology and chemical composition is available. In some species, wax platelets are dominated by high amounts of a single chemical compound, which can be primary alcohols, alkanes, aldehydes, esters, secondary alcohols, or flavonoids [26]. In contrast to platelets, plates are polygonal crystalloids with distinct edges and are attached to the surface at varying angles.

The morphology of three-dimensional wax structures is not necessarily determined by the dominating chemical compound or compound class. One example of wax crystals determined by a minor component of a complex mixture is the transversely ridged rodlets, shown in Fig. 7g, which contain high amounts of hentriacontane-16-one (palmitone) [48]. Wax rodlets are massive sculptures which are irregular, polygonal, triangular, or circular in their cross sections. They have a distinct longitudinal axis, with a length/width ratio usually not exceeding 50:1. In addition, rodlets may have a variable diameter along the length of their axis. More complex structures are the longitudinally ridged rodlets, as those found on banana leaves (*Musa* species), shown in Fig. 7h. These waxes consist exclusively of aliphatic compounds, with high amounts of wax esters and less of hydrocarbons, aldehydes, primary alcohols, and fatty acids. The origin of these wax aggregates is still not clear, and so far all attempts to recrystallize these wax types have failed. As a consequence of that, it is assumed that their origin is connected to structural properties of the underlying plant cuticle.

*Brassica oleracea* is known to have very complex wax crystal morphology, several cultivars form several different wax types, and where several different wax morphologies can occur on the same cell surface [30]. Why the different three-dimensional wax morphologies co-exist on the surface of a single cell is unknown, as is whether these different morphologies are built up by phase separation of different compounds or if they are formed by the same compound.

The last example in Fig. 7i represents plant surfaces on which waxes are arranged in a specific pattern. Examples are parallel rows of longitudinally aligned platelets, with the orientation extending over several cells (e.g., in *Convallaria majalis*, shown in Fig. 7i), or rosettes, in which the arrangements of platelets are more or less in radially assembled clusters. In particular, the parallel orientation of platelets on the leaves of several plant species leads to the question of how the orientation is controlled by the plant. It is assumed that the cutin network functions as a template for the growth of the three-dimensional wax crystals, but there is still a lack of information about the molecular structure of the cuticle, so this question is still unanswered.

Certain surface wax morphologies and their orientation patterns are characteristic for certain groups of plants; thus, patterns and the morphology of plant waxes have been used in plant systematics. Barthlott et al. [47] provide an overview of the existence of the most important wax types in plants, based on SEM analysis of at least 13,000 species, representing all major groups of vascular plants.

### Chemical Heterogeneities

Surfaces of a particular plant species may exhibit chemical heterogeneities in the classical sense, the best example are the superhydrophilic pinning anchor cells on top of each superhydrophobic trichome of *Salvinia molesta* (Fig. 28): In a broader sense, all organism have chemically heterogeneous surfaces: root surfaces differ dramatically from leaf surfaces. And within one leaf, the upper side differs from the underside. In leaves of *Quercus robur,* contact angles range from 30° to 130° depending on the part of the leaves where wettability was determined [7].

The aquatic watermilfoil *Myriophyllum brasiliense* is—like all submersed water plants—superhydrophilic, a contact angle of the leaves cannot be determined. However, as soon a flowering shoot approaches the water level, wax crystals are generated and the new leaves outside of the water exhibit a contact angle of 162° like in a lotus leaf [7].

### Crystallinity

All aliphatic plant surface waxes have a crystalline order. The classical definition for crystals implies a periodic structure in three dimensions, but with the increasing importance of liquid crystals and the detection of quasicrystals, it has become necessary to extend the definition, so that certain less periodic and helical structures, as found for some waxes, were included [49].

**Fig. 9:**
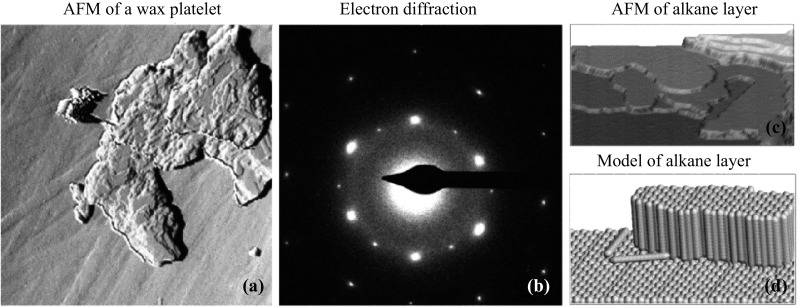
The layered and crystalline structure of alkane waxes is demonstrated by an AFM map of a single wax platelet (**a**) and the corresponding electron diffraction pattern, shown in (**b**). In (**a**) the steps visible on the crystal surface are caused by a perpendicular orientation of the molecules. Such steps can be monomolecular, e.g., for alkanes, or in some waxes bilayers are formed by polar molecules of primary alcohols. The AFM map of recrystallized alkanes (**c**) and the model shown in (**d**) demonstrate the layered orthorhombic wax structure

The crystal structure of the epicuticular waxes can be examined by electron diffraction (ED), nuclear mass resonance (NMR) spectroscopy, and X-ray powder diffraction (XRD). ED with the TEM provides the structure information of single wax crystals of less than 1 µm size, as shown in Fig. 9a, b, for a single wax platelet. However, even with a low-intensity imaging system, the crystal structure is rapidly destroyed by the electron beam intensity. Therefore, XRD is useful for determining the crystal symmetry, as well as providing information about different types of disorder. Very thin mono- or bi-molecular layers of waxes, as shown in Fig. 9c, d, are of course not periodic in three dimensions, but form two-dimensional crystals at the molecular level. As mentioned before, in addition the planar wax structures, such as films and platelets, many natural plant waxes develop irregular three-dimensional morphologies, or structures such as threads and tubules with a large extension in one direction. These morphologically different waxes were found to occur in three different crystal structures. The majority of waxes exhibit an orthorhombic structure, which is the most common for pure aliphatic compounds. Tubules containing mainly secondary alcohols show diffraction reflections of a triclinic phase, with a relatively large disorder, and ß-diketone tubules show a hexagonal structure [42].

Self-assembly of waxes is an inherent result of the crystalline nature. That different wax morphologies on plant surfaces originate by self-assembly of the wax molecules has been shown by the recrystallization of waxes, which were isolated from plant surfaces [28, 40, 45, 50–52]. In these studies, most waxes recrystallized in their original morphology, as found on the plant surfaces.

Self-assembly processes resulting in nano- and micro-structures are found in nature, as well as in engineering. They are the basis for highly efficient ways of structuring surfaces down to the molecular level. Self-assembly is a general process of structuring in which atoms, molecules, particles, or other building units interact and self-organize to form well-defined structures. The processes of self-assembly in molecular systems are determined by five characteristics: the components, interactions, reversibility, environment, and mass transport with agitation [53]. The most important driving forces are weak and non-covalent intermolecular interactions, such as Van der Waals and Coulomb interactions, hydrophobic interactions, and hydrogen bonds. During self-assembly, their interactions start from a less-ordered state, e.g., dissolved waxes in a solution, to a final more-ordered state, a crystal [54, 55]. Environmental factors such as temperature, solvent, and substrate might influence the self-assembly process, and in the case of waxes, their morphology.

**Fig. 10:**
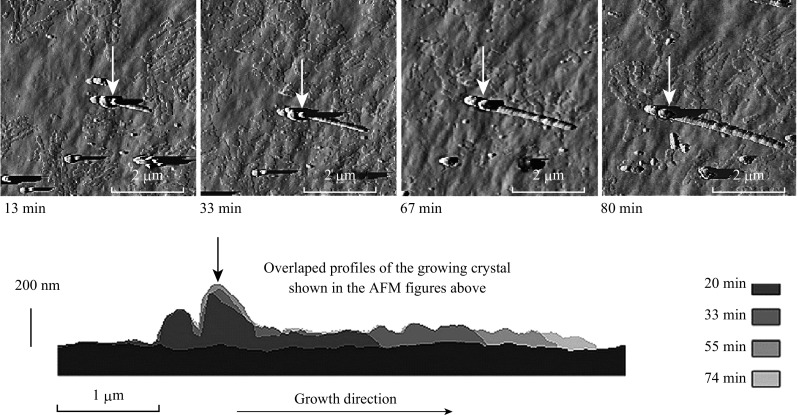
AFM maps and a series of profile lines, taken from repeated scans during the crystal growth on a leaf of Snowdrop (*Galanthus nivalis*). The first AFM map represents the wax regeneration within 13 min; the last map was taken after 80 min after wax removal. The *white arrows* mark the same position of the crystal as the *black arrow* marks in the profile figure. In the figure below, the outlines of the growing crystal have been overlapped to demonstrate, that the extension is occurring at the distal end of the growing crystal and that at this time the growth in height is limited to a few nanometers. Outlines have been taken from four AFM scans: 20, 33, 55, and 74 min after the wax regeneration process began. The experimental set-up is shown in Fig. 8

The most suitable microscopy technique for studying the self-assembly process of waxes under environmental conditions is atomic force microscopy (AFM) because it combines sufficient resolving power to image nanostructures with the ability to work at STP (standard temperature and pressure) with living plant material (Fig. 8). Self-assembly of waxes has been studied directly on plant surfaces, as well as the recrystallization of waxes and single wax compounds on artificial surfaces. However, AFM is not suitable for all plant surfaces. Within a leaf surface, large structures such as hairs with dimensions of several tens of micrometers can emerge out of the epidermis and pose a barrier against the surface scanning probe. Additionally, high aspect ratio structures caused by cell surface structures might cause artifacts in the resulting images. Species with smooth or slightly convex cell surface sculptures are most appropriate for AFM investigations. The process of wax regeneration occurs over several hours; thus the loss of water from inside the plant has to be minimized to reduce the specimen drift by material shrinking during investigation. This precondition limits the range of specimens for AFM with a small specimen chamber, because the sizes and shapes of the leaves must allow them to be mounted in the AFM without cutting them. An experimental set-up where the complete plant is placed close to the AFM and a leaf is fixed on the AFM specimen holder is shown in Fig. 8. The leaf was fixed at its lower side to the specimen holder with a drop of a two-compound glue, and waxes on the upper leaf side were removed by embedding them into a drop of water soluble glue. After hardening, the glue and the embedded waxes were removed from the leaf surface and the process of wax regeneration was studied. Temperature increase in long-term investigations, caused by the laser beam on top of the cantilever, induces expansion of the water in the leaf, resulting in a drift of the specimen. To minimize this, reflective cantilevers must be used, and the laser beam intensity should be reduced by integrating an attenuation filter above the cantilever [40]. However, the waxes themselves are fragile; thus appropriate scan conditions at scan sizes of 3–20 µm are tapping mode and scan rates of 0.7–2 Hz, encompassing 256 lines per image and a set-point near the upper limit to minimize the interaction between tip and sample. Figure 10 shows the regeneration of a wax film on a leaf of snowdrop (*Galanthus nivalis*) by formation of a multilayered wax film and the growth of three-dimensional wax platelets. This and further investigations show that the growth of the three-dimensional wax crystals occurs by apical accumulation of new wax molecules on only one side of the crystal. The regeneration of the wax film results in a multilayered crystalline coverage on the plant cuticle. The time needed to regenerate waxes shows large variations depending on the species, with some species never regenerating the wax that was removed. In these plants, wax synthesis seems to be inactive when leaves are mature [56].

**Fig. 11:**
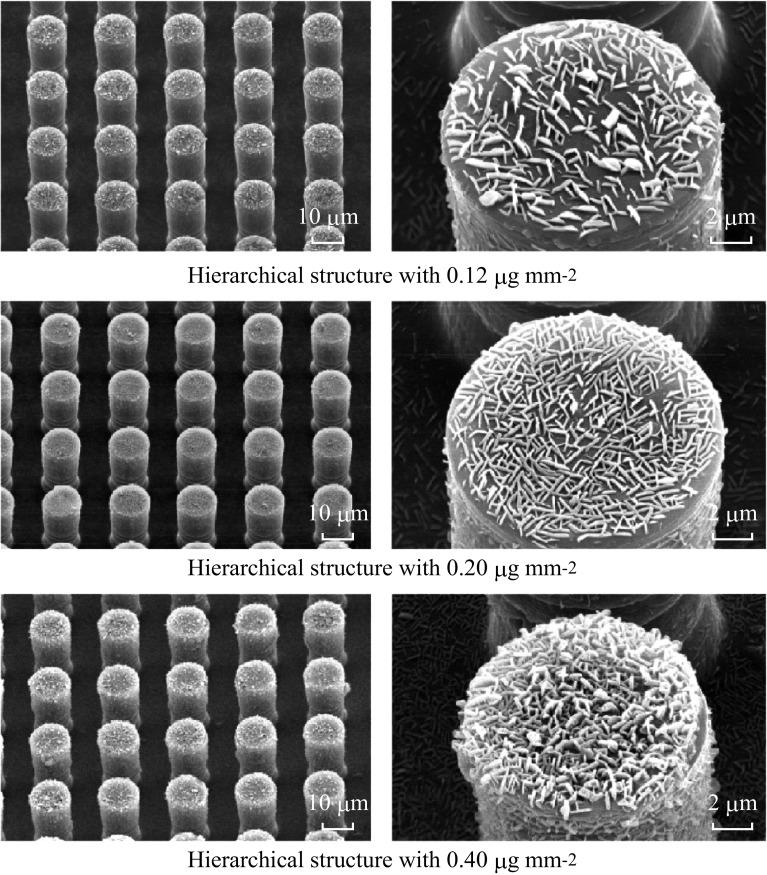
Biomimetic superhydrophobic hierarchically structured technical surfaces. The silicon replica with pillars of 14 μm in diameter and 30 μm in height with 23 μm pitch, covered by self-assembled alkanes (hexatriacontane). From *top* to *bottom* an increase in crystal density is shown. Highest water repellence and lowest hysteresis has been found for the structures given in the middle line, where 20 µg cm^−2^ hexatriacontane was applied on the surfaces. These surfaces have been used for detailed study of wetting and adhesion (from Ref. [59])

**Fig. 12:**
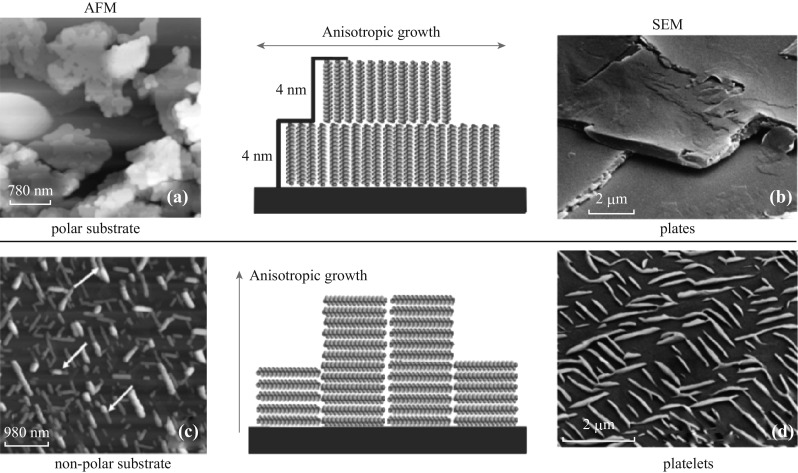
AFM maps and schematics of the molecular orientation demonstrate the differences of growth on polar and non-polar substrates for octacosan-1-ol molecules. AFM figures (**a**, **c**) show growing crystals, whereas the SEM figures (**b**, **d**) show the final crystal morphology. On both substrates flat crystals with different orientations were grown. Crystals grown parallel to the substrate surface, as shown in (**a**, **b**), are called plates; crystals grown perpendicular to the substrate surface are called platelets (**c**, **d**). The principle of anisotropic crystal growth is shown schematically for both preferred growth directions and described in the text

Alternatively, self-assembly of plant waxes can be studied by recrystallization of the waxes on artificial substrates (Fig. 11). Based on those studies, the formation of wax tubules and platelets has been described in detail. Wax platelets, characteristic for wheat leaves (*Triticum aestivum*), are constructed from the primary alcohol octacosan-1-ol [28]. Crystallization of the wax mixture isolated from the leaves and of pure octacosan-1-ol on different artificial substrates showed a substrate-dependent growth. On a non-polar, crystalline substrate (highly ordered pyrolytic graphite, HOPG), platelets grow with a vertical orientation to the substrates, whereas on a polar surface, such as mica, crystals grow horizontally to the substrate surface. On amorphous polar glass only amorphous wax layers grow. This substrate dependence demonstrates epitaxial control of crystal growth depending on the orientation and order of the first layers of molecules adhering on the substrate surface. Octacosan-1-ol forms ordered bilayer structures on the substrate. In these, the first layers of molecules lie flat on non-polar substrates, but stand upright (perpendicular) on crystalline polar surfaces. The grown platelet morphology results from an anisotropic crystal growth, caused by a faster parallel assembly of the molecules at the length side of already existing molecules than at the ends of the molecules [57]. AFM micrographs in Fig. 12 and schematics of the molecule orientation demonstrate the differences of growth on polar and non-polar substrates for octacosan-1-ol molecules. In both cases, flat crystals with different orientations grow. Crystals grown horizontal to the substrate surface are called plates (Fig. 12a, b), those grown perpendicular to the substrate surface are termed platelets (Fig. 12c, d). The substrates on which the crystals grow influence the crystal morphology and their orientation. This fact can be used to create different kinds of nano- and micro-patterns on technical surfaces [28, 52, 58, 59]. In summary, substrates can have a direct influence on the self-assembly processes of wax crystals, and can function as a template on the molecular level. In this case, the substrate organizes the assembly of the molecules in a specific spatial arrangement [60, 61]. Such a template effect was reported for wax platelets formed by primary alcohols [28]. On HOPG substrate, the spatial pattern of the reassembled wax platelets strictly followed the hexagonal symmetry of the crystalline substrate. However, the cutin matrix of the cuticle, which acts as a substrate in plant surfaces, is assumed to be amorphous, and an epitaxial growth on an amorphous substrate seems paradoxical.

An example of wax crystals composed of more than one compound is the transversely ridged rodlets. These waxes can be recrystallized from the total wax mixture, but not from individual compounds such as alkanes or palmitones. For these waxes, it is assumed that their morphologies are also formed by a self-assembly-based crystallization process, but the presence of minor amounts of other compounds is required as an additive for crystal growth [48].

**Fig. 13:**
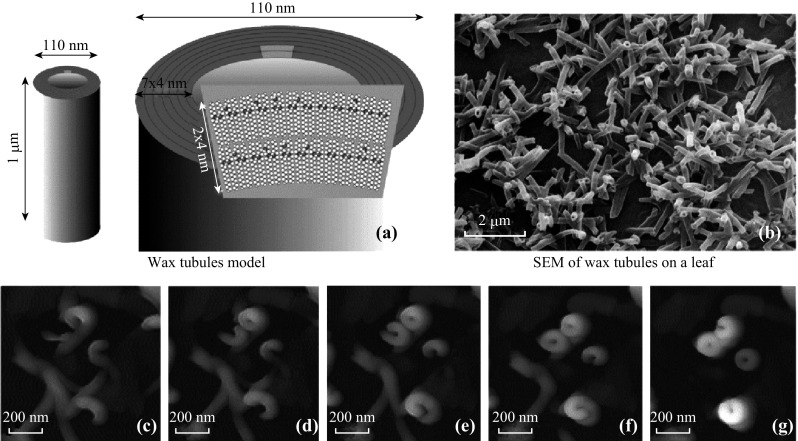
A model and SEM micrograph of the molecular order of nonacosan-10-ol tubules and AFM analysis of their self-assembly. Based on SEM characterization, chemical analysis, single compound crystallization, and crystallographic data, a model of the nonacosanol tubules has been developed (**a**). Original nonacosan-10-ol tubules are shown in the SEM micrograph (**b**) for *Thalictrum flavum glaucum* leaves. Consecutive AFM figures of tubule formation (nonacosan-10-ol wax from *Tropaeolum majus*) were made after applying a wax solution on HOPG. After 65 min (**c**) the waxes mainly formed curved rodlets, which were horizontally arranged to the substrate. The same area of the HOPG substrate shows that waxes start to form circles (**d**–**g**) and after 223 min (**g**) the rodlets initially observed were dissolved and short tubules were formed

The origin of wax tubules, shown schematically in Fig. 13a and in SEM micrographs in Fig. 13b, has been debated for a long time. Several observations, such as spiral lines on the surfaces of some nonacosanol tubules [51], led to the assumption that tubules arise from a twisting or folding of a platelet-like precursor form. Recrystallization experiments with nonacosan-10-ol waxes showed that these tubules grow perpendicular to the substrate surface when recrystallized on HOPG. This vertical orientation of the tubules allows a detailed study of the growth process by AFM and shows that the building of nonacosan-10-ol tubules from Lotus (*Nelumbo nucifera*) and Nasturtium (*Tropaeolum majus*) leaves is based on a continuous growth of a small circular precursor structure by supplementation of the wax on top of it [52]. The AFM micrographs shown in Fig. 13c–g, are consecutive AFM images of growing tubules, made during the tubule formation process. The terminal ends of growing tubules are asymmetric in height. This asymmetry seems to be caused by an accumulation of new wax molecules at edges found at the terminal end of the tubules and indicates a helical growth mechanism for the tubules. The pure nonacosan-10-ol alcohol, the dominating compound of wax tubules, can crystallize in different forms [39, 45, 51]. Here, Jetter and Riederer [45] show that a range of alkanediols, present in the waxes of many secondary alcohol tube-forming species, also have tube-forming capability.

Chemical analysis of the leaf waxes of Lotus and Nasturtium (*Tropaeolum majus*) shows that waxes of both species are composed of a mixture of aliphatic compounds, with nonacosan-10-ol (a secondary alcohol) and nonacosandiols (an C_29_ alkane with two alcohol groups) as their main components [52]. These compounds have been separated from the rest of the wax compounds and used for recrystallization experiments. It could be shown with mixtures of nonacosan-10-ol and nonacosandiols components that a minimum amount of two percent of nonacosandiols support tubules formation [23].

Analysis of wax chemistry, crystalline order, and their self-assembly has led to a better understanding of the molecular architecture of three-dimensional waxes [62]. Based on these data, a model of nonacosan-10-ol tubule structure has been developed, as shown in Fig. 13a. Here it is assumed that the lateral oxygen atoms at the side of the straight molecules hinder the formation of the normal, densely packed, orthorhombic structure and require additional space, causing a local disorder between the molecules and cause a spiral growth, leading to the tubule form.

Glands or glandular trichomes may produce very particular substances, they can be found on approximately 30% of all vascular plants [63]. Multicellular glands include salt glands, nectaries, or the adhesive-secreting glands of some carnivorous plants [64]. Secretion and accumulation of toxic compounds at the plant surface allows direct contact with insects, pathogens, and herbivores, and might therefore be an effective defense strategy [64]. The exudates of glands are, for example, terpenoids, nicotine, alkaloids, or flavonoids. The exudates of some ferns and angiosperms, in particular several members of the *Primulaceae*, are composed of flavonoids [65, 66]. These flavonoid exudates or “farina” are morphologically similar to waxes, but are chemically distinct from plant waxes. Other glandular trichomes, such as the glands of the carnivorous plants of the genera *Drosera* (sundew) and *Pinguicula* (butterworts) secrete adhesives and enzymes to trap and digest small insects like mosquitoes and fruit flies.

Chemical heterogeneities are implied by the presence of other glands. The definition of this phenomenon depends on the scale: all biological surfaces show chemical heterogeneities, the most common case are leaves with a hydrophilic upper side and a superhydrophobic lower side. On a much smaller scale, the trichomes of certain Salvinia species are most remarkable for hydrophilic islands within a superhydrophobic surface, the Salvinia paradox (Fig. 28) [1, 67].

## Structuring of Plant Surfaces: Hierarchical Architecture between Nano- and Macrostructures

### The Cuticle

Primarily water plants (from unicellular algae to giant seaweeds) lack a cuticle; this particular polymer layer is restricted to higher plants (see Sect. 8).

The cuticle covers leaves, flowers, stems, fruits, and seeds and serves as a protective continuous layer covering the primary organs of all vascular plants and mosses. But in roots and secondary structures (e.g., bark) a cuticle is not present. The cuticle is a hydrophobic composite material, composed of a polymer called cutin and integrated and superimposed lipids called “waxes” (see Sect. 2). The cuticle network is formed by cutin, a polyester-like biopolymer composed of hydroxyl and hydroxyepoxy fatty acids, and sometimes also by cutan, which is built by polymethylene chains. Non-lipid compounds of the cuticle are cellulose, pectin, phenols, and proteins. Large differences in the chemical composition and microstructure of the cuticle have been found by comparing different species and different developmental stages. Chemical composition, microstructure, and biosynthesis of the cuticle have been reviewed by several authors [26, 68–75].

### Hierarchical Sculpturing

**Fig. 14:**
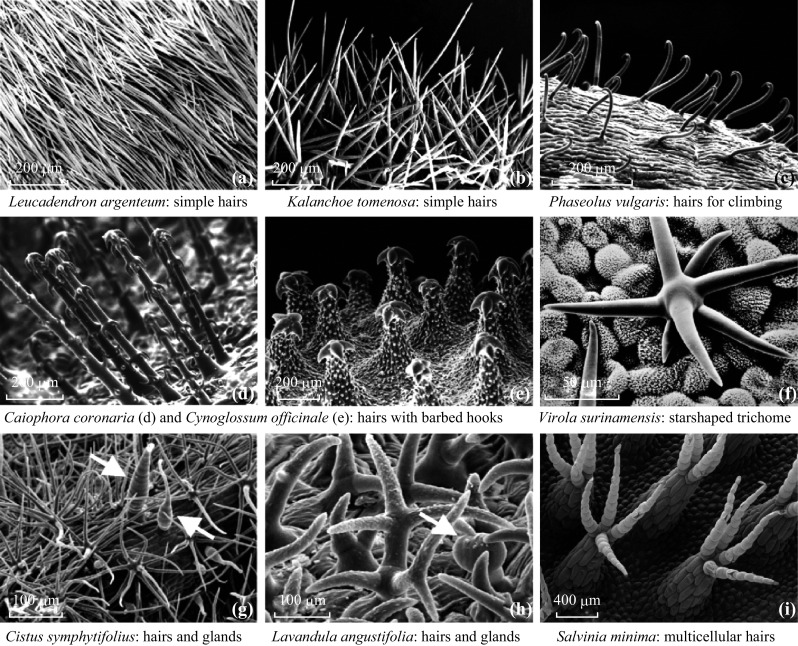
SEM micrographs of hairs and glands on plant surfaces. **a** A dense layer of straight, unbranched hairs almost orientated parallel to the leaf surface on *Leucadendron argenteum*. **b** The unbranched hairs of *Kalanchoe tomentosa* are orientated upright. **c** The shoot surface of a bean shoot *Phaseolus vulgaris* with terminal hooks to facilitate climbing. **d** Single hairs on a leaf of *Caiophora coronaria* and **e** those on the seed surface of *Cynoglossum officinale* are characterized by terminal and lateral barbed hooks. **f** The star-like trichome on the leaf of *Virola surinamensis* has a flat surface in contrast to the convex epidermal cells with epicuticular wax crystalloids. **g** Simple branched star-like hairs and two morphological different glands (*arrows*) on the leaf of *Cistus symphytifolius*, and **h** multiple ramified hairs and short-stalked glands (*arrow*) on a leaf of *Lavandula angustifolia*. **i** The four trichomes of *Salvinia minima* originate from a common base, in contrast to *S. molesta* (Fig. 28) the tips are free and do not show the *eggbeater shape*

The cuticle and molecular wax films are the foundation of the surface. Additional levels of “hierarchical sculpturing” (or less precise “structuring”) are formed by wax crystals (Fig. 7), the form of single cells (Fig. 18), composed of sculptures like simple or multicellular hairs (Fig. 14), the form and curvature of whole organs likes leaves (Fig. 27b, c) up to the gross morphological levels visible from larger distances like the riblets of the Titan arum flower (*Amorphophallus,* Fig. 1). The concept of hierarchical sculpturing of plant surfaces and a coherent terminology were introduced by Ref. [2] and used in many publications (e.g., Ref. [5, 76]). Based on the comprehensive survey by Ref. [1], we have revised this concept starting with a “flat” surface as first level, followed by a sequence of 4 or more superimposed “micro-architectural” hierarchical levels. The term *sculpture* (“architecture”) or sculptural seems more appropriate for three-dimensional features (discussed previously in Ref. [2]). *The terms structure* or *structural* also includes chemistry and chemical heterogeneities, we prefer thus to use the term “sculpture” for the micro-architectural elements within the following four levels:

### First Sculptural Level

Flat surfaces defined by their hydrophilic or hydrophobic chemistry on the scale of the resolution of a scanning electron microscope (SEM). Flat is a relative category that depends on the scale. Here, we limit the definition of flat to surfaces that feature structures of usually less than 10 nm in height. Flat surfaces are rarely found in plants and animals, e.g., the leaves of Rubber Figs (*Ficus elastic*).

### Second Sculptural Level

Cell surfaces are covered with structures between 50 nm and 20 µm. Structures at this level on plants are usually formed by epicuticular wax crystals (e.g., Fig. 7) which may exhibit a large spectrum of shapes like rodlets, platelets, or tubules. They may exceed 200 µm in height (e.g., *Strelitzia, Copernicia, Benincasa*).

**Fig. 15:**
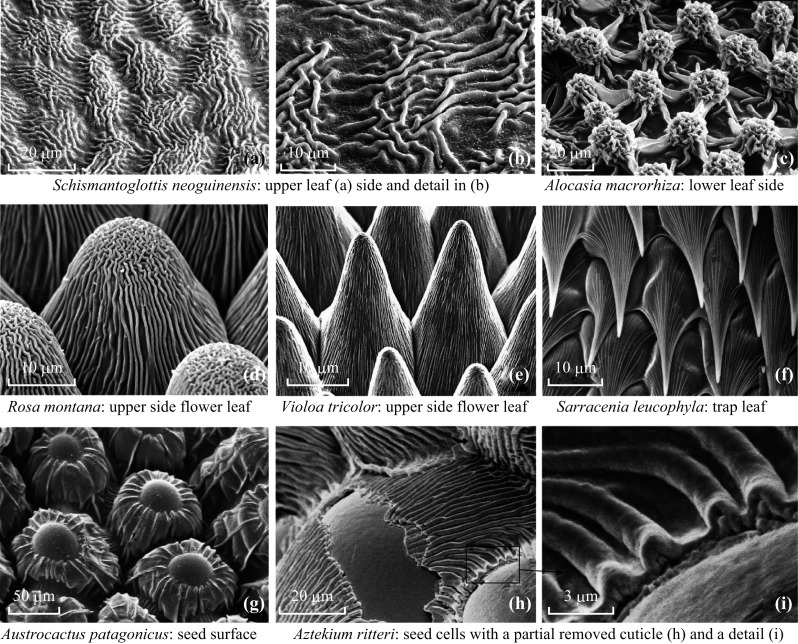
SEM micrographs of cell surfaces with cuticular folding. **a**, **b** The irregular cuticular folding on a leaf of *Schismatoglottis neoguinensis* is restricted to the central field of the cells. **c** *Alocasia macrorrhiza,* the cells are flat (tabular), with nodes like exposed central fields of the cells. **d** The cells of a flower petal of *Rosa montana* with a rippled-folded cuticle in the central field of the cells and parallel folding, running to the anticlinal walls of the cells. **e** Conical cells of the flower petals of *Viola tricolor* with parallel folding. **f** The cells of the inner side of a tube-like leaf of the carnivorous plant *Sarracenia leucophylla*. These cells have a conical hair-papilla in the downward direction with a parallel cuticle folding, with larger distance at the base and denser arrangement at the cell tip. **g**–**i** seed surfaces. **g** In *Austrocactus patagonicus*, the central field of the cells is unstructured, whereas a rough folding exists in the anticlinal fields. **h**, **i** In *Aztekium ritteri*, a part of the cuticle has been removed to show the cuticle folding

**Fig. 16:**
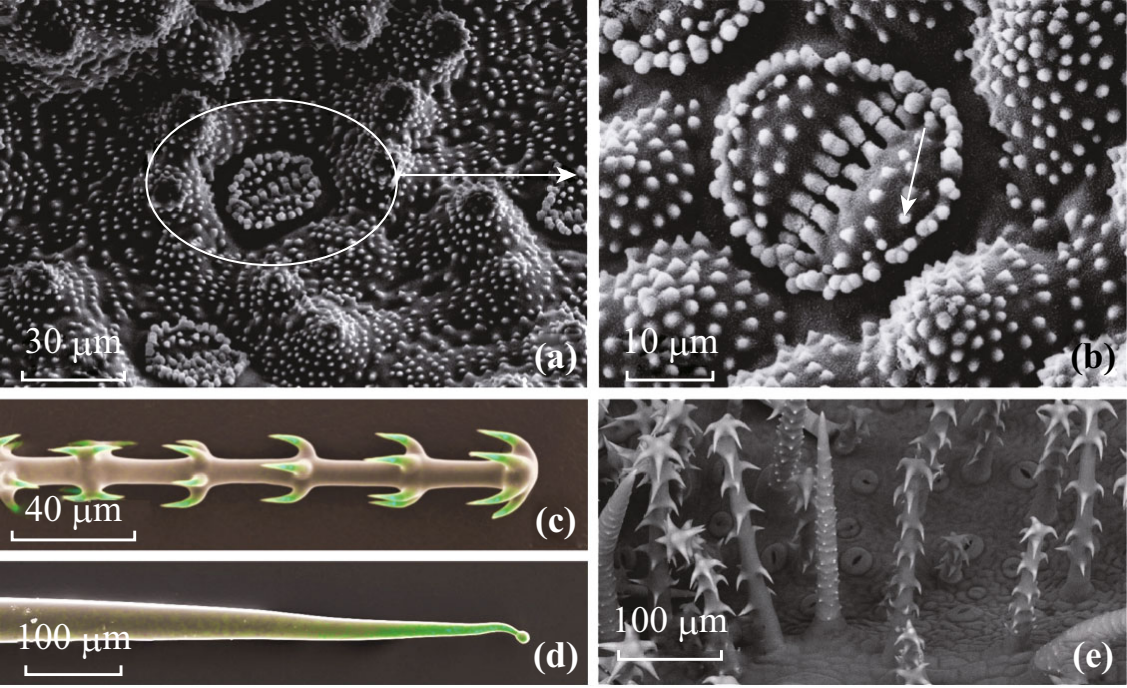
Cell surface structuring by sub-cuticular silicon dioxide and apatite insertions. **a**, **b** SEM micrographs of the horsetail (*Equisetum arvense*). **b** A detail of (**a**) shows that the stomata and their surrounding cells have a micro-pattern of small enhanced spots, formed by sub-cuticular inserts of silicon-oxide crystals. **c**, **d**, **e** Complex glochidia hooked (**e**) and stinging (**d**) hairs are shown on the leaf surface in the flower nettle *Loasa*. The trichomes of Loasaceae are unique and mineralized by apatite. **c**, **e** from Ref. [80]

**Fig. 17:**
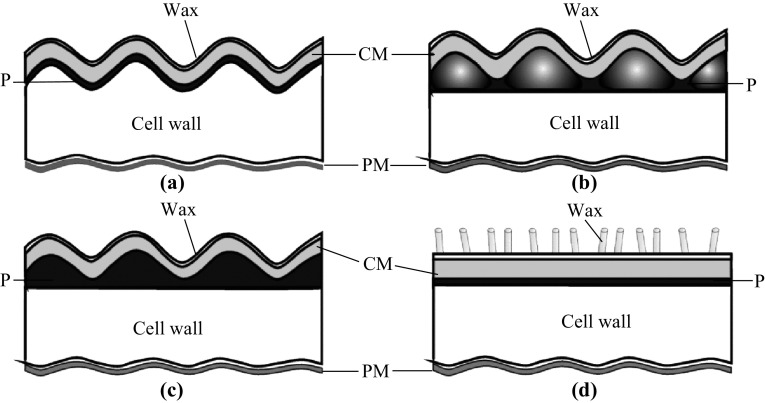
Schematic of cross sections through plant epidermis cells show different sources leading to micro-structuring of cell surfaces. In (**a**) the surface profile is induced by coves of the underlying cell wall, in (**b**) by insertion of sub-cuticular minerals, in (**c**) by folding of the cuticle and in (**d**) by waxes, which are located on top of the cuticle (epicuticular wax). *Wax* epicuticular waxes, *CM* cuticular membrane, *P* pectin, *PM* plasma membrane (modified after Ref. [2])

The main elements of the second sculptural level in plants are (i) epicuticular wax crystals, the diversity of waxes was discussed in the preceding chapter on the chemistry of surfaces, (ii) cuticular folds (Fig. 15), (iii) sub-cuticular inclusions (Fig. 16). Functionally, a superimposition of cuticular folds and wax crystals is not necessary: Structuring on a specific level is performed by one group of elements, which seems to be a basic law [2]. The data gathered by investigating thousands of plant surfaces constitute the rule that, e.g., wax crystals and cuticular folds exclude each other. These frequently found morphological modifications of the outermost cell walls are known to influence the second hierarchical level. They are schematically shown in Fig. 17. In the first case, shown in Fig. 17a, the surface structure is induced by concavities of the cell wall which lead to coves and folding of the surface. The second kind of structuring originates by sub-cuticular inserts of mineral crystals, such as silicon oxides (Fig. 17b). The third kind of surface structuring results from the folding of the cuticle itself (Fig. 17c). Additionally, on many plants, waxes on top of the cuticle lead to surface structuring as shown in Fig. 17d. Waxes and their structural diversity have already been introduced; thus, cuticular folds and sub-cuticular are introduced in the next chapter.

Cuticular foldings have been described for nearly all epidermal surfaces of plants, but are frequently found in the leaves of flowers (petals), and on seed surfaces. They occur as folding or tubercular (verrucate) patterns, which originate due to the cuticle itself by an overproduction of cutin [77]. The pattern of cuticular folds can be categorized according to the thickness (width) of the folds, distances between the folds, and by their orientation [2]. Additionally, the pattern of folding within a single cell can be different in the central (inner area) and anticlinal field (outer area) of a cell. Figure 15 shows different patterns of cuticle folding. On the leaves of *Schismatoglottis neoguinensis* (Fig. 15a, b) the folding is orderless and covers the central and anticlinal field of the cells. On the lower leaf side (adaxial) of *Alocasia macrorrhiza,* shown in Fig. 15c, the cuticle forms node-like folding in the central part of each cell. The flower petals of *Rosa montana,* shown in Fig. 15d, have convex cells with a small central field with a rippled-folded cuticle and parallel folds in the anticlinal field. The papilla cells of the flower petals of V*iola tricolor,* shown in Fig. 15e, have a parallel folding from the center to the anticlines of the cells. The cells inside the trap of the carnivorous plant *Sarracenia leucophylla,* shown in Fig. 15f, are hair-papillae, with a conical shape curved in a downward direction. On these, a parallel cuticle folding exists with larger distance at the base and a denser arrangement at the cell tip. The seed surface of *Austrocactus patagonicus,* shown in Fig. 15g, has cupular formed cells with unstructured central fields and broad parallel folds in the anticlinal fields. A high-magnification SEM micrograph of the seed surface of *Aztekium ritteri,* shown in Fig. 15h, i, shows a partially removed cuticle and demonstrates that the origin of surface folding is caused by the cuticle itself.

Some micro-structures on epidermis cells arise from sub-cuticular inserts of mineral crystals, as indicated in Fig. 17b. These sub-cuticular inserts can be solid crystals of silicon dioxide, as shown in Fig. 16a, b for tin plant or horse tail (*Equisetum*) plants. Calcium oxalate crystals are also frequently found in plants, and verification of silicon or calcium presence can be made simply by energy dispersive X-rays (EDX) analysis, included in SEM. Silicon (Si) is a bioactive element associated with beneficial effects on mechanical and physiological properties of plants. It is a common element found in plants and occurs as monosilic acid or in the polymerized form as phytoliths (SiO_2_–*n*H_2_O) [78]. In plants, Silica tends to crystallize in the form of silica in cell walls, cell lumina, at intercellular spaces and in the sub-cuticular layer [79]. Recently Ensikat et al. [80] investigated calcium apatite, a material which plays a crucial role in animals, in the complex trichomes of the family Loasaceae (Fig. 16c–e). Calcium oxalate crystals have been reported for more than 250 plant families [81]; they are deposed within the living tissue.

### Third Sculptural Level

Unicellular (multicellular in certain hair types in plants) structures usually caused by particular shapes of the outer cell wall which may vary from convex to papillose cells and ultimately to hairs, which may be unicellular or multicellular (for a terminology see Ref. [2]); dimensions range from about 2 µm to several centimeters, i.e., in trichomes, (hairs). Structures of the second level may be superimposed to structures of the third level (e.g., Fig. 22). To understand this level, often a thorough microscopic analysis is essential, as the description and terminology for this diversity are complex.

*The outlines of cells.* The description of plant micro- and nanostructures requires the use of some basic uniform terms, for example, to describe the outline of a single epidermis cell. Several variations are known and introduced in detail by Barthlott and Ehler [2], Barthlott [76], and Koch et al. [82, 83]. In the following, a brief introduction is given.

**Fig. 18:**
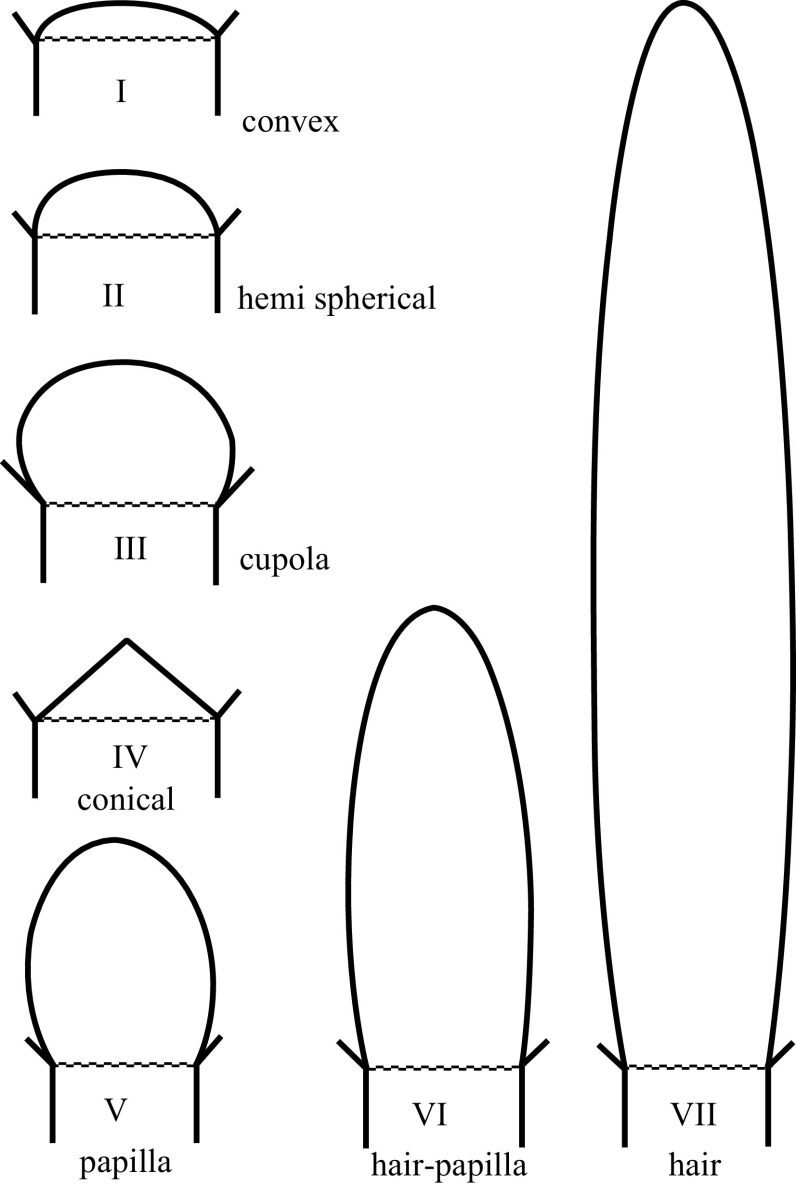
A schematic of different convex cell outlines and their aspect ratios (ß = width/height) of the convex cell types and their terminology: convex (ß > 3/1), hemispherical (ß ~ 2/1), cupola (ß < 3/2), conical (ß > 3/2), papilla (ß < 3/2 and > 1/2), hair-papilla (ß < 1/3 and > 1/6), and hair (ß < 1/7)

The boundary walls between two adjacent epidermal cells are called anticlinal walls, whereas the outer wall forming the cell surface is called the periclinal wall. The primary sculpture of a single cell encompasses the outline, including the shape and relief of the anticlines and curvature of the outer periclinal wall. There are two basic forms of cells, the tetragonal and polygonal form, both of which can have a uniform length of their sides or be elongated. Additionally, the course of the anticlinal walls can be straight or uneven. It is assumed that the outline of anticlines has an influence on the mechanical stability of the epidermis tissue, but experimental evidence for this hypothesis is not available. The cell sculptures or curvature of the outer epidermis wall (periclinal wall) can be tabular (flat), convex (arced to the outside), or concave (arced to the inside), and have a large influence on the surface roughness at the micrometer scale. Additionally, only the central area of a cell can form a convex outgrowth and form a papilla or hair-like structure. The convex cell type is the most common cell type of epidermal surfaces, often found on flower leaves, stems, and leaves [84]. These cell morphologies originate by the expansion of the outer side (periclinal wall) of the epidermis cells. They can be divided into several sub-types, depending on the outline of the epidermis cells and their aspect ratio (width to height), which determines their designation. In Fig. 18, a schematic of different convex cell outlines and their designations is given. The terminology is based on the cell outline and aspect ratios (ß = width/height) of the cells and includes: convex (ß ≥ 3/1), hemispherical (ß ~ 2/1), cupola (ß < 3/2), conical (ß > 3/2), papilla (ß < 3/2 and > 1/2), hair-papilla (ß < 1/3 and > 1/6), and hairs (ß < 1/7). In these, hairs are built by the outgrowth of a single surface cell. Hairs are often named trichome (gr.: trichoma).

The leaf surfaces of *Leucadendron argenteum* and *Kalanchoe tomentosa,* shown in the SEM micrographs in Figs. 14a, b, are two representative surfaces with hairs. Hairs can decrease, but also increase the loss of water and influence the wettability of the surfaces [85]. The wide spectrum of functions of plant hairs has been reviewed by Wagner et al. [64], and more recently by Martin and Glover [84]. With respect to their functions, it is important to notice that hairs can be glandular or non-glandular (non-secreting), dead or living, and hairs can also be built up by several cells (multicellular), which are introduced later. Unicellular trichomes can be found on the aerial surfaces of most flower-plants (angiosperms), some conifers (gymnosperms) and on some mosses (bryophytes) [64]. Many plants of dry habitats show a dense cover of dead, air-filled hairs to reflect the visible light, which makes the surfaces appear white. The structures of hairs are often more complex; thus, the definition based on the aspect ratio fits well only for simple, undivided hairs. On the shoots of common beans (*Phaseolus vulgaris*), hairs form hooks to get better adhesion for climbing (Fig. 14c) and in *Caiophora coronaria* (Fig. 14d) and *Cynoglossum officinale* (Fig. 14e) the hairs have lateral barbed hooks. The stellate hairs of *Virola surinamensis* differ by having completely smooth surfaces from the other epidermal cells covered by a dense layer of wax crystals (Fig. 14f). Further trichomes are the simple or double-branched hairs and secretion glands on the leaves of *Cistus symphytifolius* and *Lavandula angustifolia* as shown in Fig. 14g, h. These complex hair structures require a more differentiated description than the aspect ratio used for simple hairs [86, 87]. The sizes and morphologies of trichomes are often species specific, making some trichomes useful as morphological features in plant systematics [87]. Deformation induced by water loss of dead-desiccated cells can leads to concave cell morphologies and other complex modifications (Fig. 3). This is characteristic for seed coats and can result in most complex hierarchical sculpturing formed in cacti [88] or orchids [89].

### Fourth Sculptural Level

Multicellular structures caused by specific arrangement patterns of several of epidermal cells. There is a wide variety of possibilities for this group of structuring. Multicellular hairs are common in all groups of vascular plants, apart from conifers.

Particularly interesting forms occur in the floating ferns of the genus *Salvinia*. Within this genus, morphologically different kinds of water-repellent (superhydrophobic) hairs exist [90], which in some species (*S. auriculata, S. molesta*) show functionally important chemical heterogeneities (see Sect. 2) by utilizing hydrophilic anchor cells at the tip of the trichomes to stabilize the air–water interfaces [1, 67]. Four different hair types have been described for the genus *Salvinia* [90]. Based on these morphological types, the genus Salvinia is divided into four groups, each with several species. The Cucullata-type is characterized by solitary and slightly bent trichomes and occurs in *S. cucullata* and *S. hastate.* The Oblongifolia-type forms groups of two trichomes, which bend in the same direction and sit on an emergence. This type occurs on *S. oblongifolia.* The Natans-type, shown in Fig. 14i, has four trichome branches, each elevated on a large multicellular base and in total has a height of up to 1300 µm. The heights of the trichome-groups decrease towards the leaf margins. This type occurs in *S. natans* and *S. minima*. In the Molesta-type (Fig. 23c), four trichome branches are grouped together, connected with each other by their terminal cells and sitting on a large emergence. The heights of these trichomes reach up to 2200 µm in *S. molesta,* but also decrease towards the leaf margins. This trichome-type is characteristic for, e.g., *S. molesta* and *S. biloba*. In all these species, the epidermis is covered with small three-dimensional waxes in the form of transversely ridged rodlets. The development of these complex structures has been studied in *S. biloba*, by Barthlott et al. [90]. In an early stage of leaf development, the hair formation starts with a grouping of four cells. During the ontogeny of the leaf, four branches develop from these initial cells and form a crown-like structure, in which the single branches are connected with each other. Later, the base grows by cell division and cell expansion to develop a large base below the crown structure.

## Physical Basis of Surface Wetting

Wetting is the fundamental process of liquid interaction at solid–gaseous interfaces. It describes how a liquid comes in contact with a solid surface. The basics of surface wetting are summarized here; extensive literature on the topic exists: Israelachvili [91], Bhushan [92], De Gennes et al. [93] and Bhushan [94], Nosonovsky and Bhushan [95], Bormashenko [96], Butt et al. [97], Schellenberger et al. [98], and Bhushan [99].

**Fig. 19:**
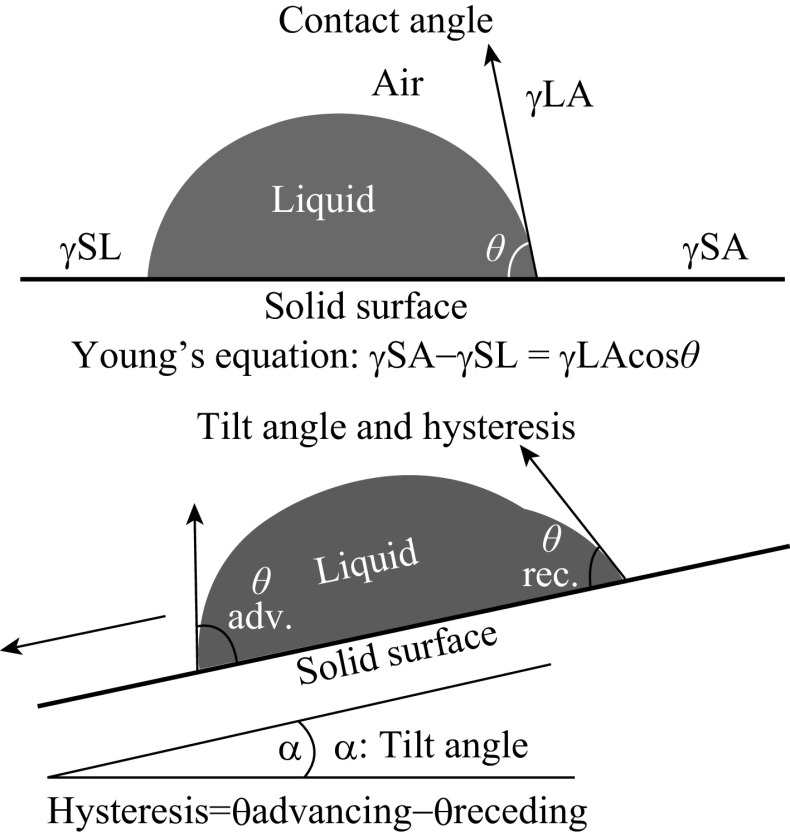
The schematic shows the wetting of a solid surface. γ_LA_, γ_LS_, and γ_SA_ are the interfacial tensions at the boundaries between liquid (L), solid (S), and air (A), which determine the CA of an applied water droplet and is described by Young`s equation. The hysteresis of a water droplet on a tilted surface represents the adhesion of the liquid on the surface and can be determined by measuring the tilting angle or the advanced and receding angle of a water droplet

Static wetting processes. A droplet on a solid surface wets the surface depending on the chemical properties of the phases, as well as on the surface structure. A parameter to quantify this wettability is the contact angle (CA). A high contact angle describes surfaces on which a water droplet forms a spherical shape and the contact area between the liquid and the solid is low. Contact angle measurement is the main method for the characterization of the hydrophobicity or hydrophilicity of surfaces. The wetting behavior of solid surfaces can be divided into four classes, defined by their CA, as well as CA-hysteresis for the case of superhydrophobic surfaces (Fig. 19). On superhydrophilic surfaces, a fluid will spread and cover a larger area of the surface, the CA ranges from 0° to <10°. Surfaces with a CA between >10° and <90° are termed hydrophilic surfaces. Un-wettable surfaces have high contact angles, meaning the liquid on the surface forms a semispherical or spherical droplet. Surfaces on which the CA is >90° and <150° are hydrophobic surfaces. A superhydrophobic surface is defined as the one that has a static CA of >150°, and if those superhydrophobic surfaces have a low hysteresis or a low tilting angle (TA) of less than 10° they are superhydrophobic and can have self-cleaning properties. This definition of superhydrophobic surfaces has been used in many reviews [96, 100–104] and represents the most used classification of surface wettability. The basis for studying the wettability of smooth surfaces is given by Young’s equation [105]. The CA of a liquid on a surface depends on the surface tension (molecular forces) of the specific liquid, solid surface, and the surrounding gas. Thus, wetting depends on the ratio between the energy necessary for the enlargement of the surface and the gain of energy due to adsorption (Fig. 19) [91, 106, 107]. The basis for studying equilibrium wetting on rough surfaces was established many years ago by Wenzel [108], Cassie and Baxter [109, 110]. The Wenzel equation expresses a general amplification of the wettability induced by roughness and applies to a CA where droplets are in equilibrium, but not to advancing and receding angles of a droplet on a rough solid surface that give rise to contact angle hysteresis (CAH). Hysteresis is responsible for the sticking of liquids on a surface, and is the difference of an advancing and receding angle of a moving droplet (CAH = CA_adv_ – CA_rec_, shown in Fig. 19). If a droplet moves over a solid surface, the CA at the front of the droplet (advancing CA) is greater than that at the back of the droplet (receding CA). However, if the droplet rolls with little resistance, the contact angle hysteresis is small. Advancing and receding contact angles can also be determined when additional liquid is added to a sessile drop and the contact line advances; if liquid is removed from the drop, the CA decreases to a receding value before the contact retreats.

On water-repellent surfaces, a droplet applied starts to roll-off the surface when it is tilted to a specific angle. This tilt angle (TA) is simply defined as the tilting angle of a surface on which an applied drop of water starts to move. Low TA (<10°) is characteristic for superhydrophobic and self-cleaning surfaces. If the droplet is in Cassie–Baxter stage [109], with air trapped between the surface and the applied water droplet, the real contact between the droplet and the surface is very small compared to wettable surfaces, on which an applied drop of water tends to spread, and contact angle is low. In intermediate wetting stages [111], high contact angles correspond with an increased contact between the surface and the water droplet applied. This phenomenon has also been described as petal-effect [107, 112] because of its occurrence in the flower leaves (petals) of some roses.

Dynamic interactions are bouncing, splashing, and spreading of droplets. The impact behavior of falling drops has been subject to studies for several liquids, such as water, ethanol, emulsions, and various types of structured and unstructured, solid and liquid surfaces [113, 114]. In addition to the physicochemical surface properties and structures, other factors determine the impact behavior of falling drops: their velocity, size, surface tension, and viscosity [115–117]. Falling drops can impact without any breakup or impact with a splash or splashing, in which the impacting drop releases smaller droplets flying away from the point of impact. Roughly two different types of splashing on incompliant surfaces are described, the “prompt splash” and the “corona splash”. The “corona splash” consists of relatively large droplets and is the outcome of breaking up fingers developing at a flattening drop’s rim. In “prompt splash” very small and fast droplets are generated when the advancing lamella of a spreading drop is disturbed by rough surface structures higher than a specific fraction of the lamella, causing the lamella to rupture locally and release small splash droplets [118].

Dynamic interactions between water droplets and the surfaces are well researched in the different types of splash-phenomena described, e.g., in Rioboo et al. [119], Yarin [113], Xu et al. [118], Motzkus et al. [120], Gilet and Bourouiba [121], and Koch and Grichnik [122]. More recently in focus came the contact times between droplets and surfaces: the time span a drop impacting and bouncing on a hydrophobic surface is in contact with the surface. The contact time as well as the shape of the rebound droplet strongly depends on the structure and the chemistry of the surface. A reduction of this contact time is of advantage in several applications, e.g., anti-icing, self-cleaning, or spray cooling [123]. But also in biological surfaces, the contact time might be important.

Dynamic wetting processes and their control may play an underestimated role in the interaction between plant surfaces and rain, the maintenance of air layers under water, or pesticide applications.

## Superhydrophilic and Superhydrophobic Plant Surfaces

**Fig. 20:**
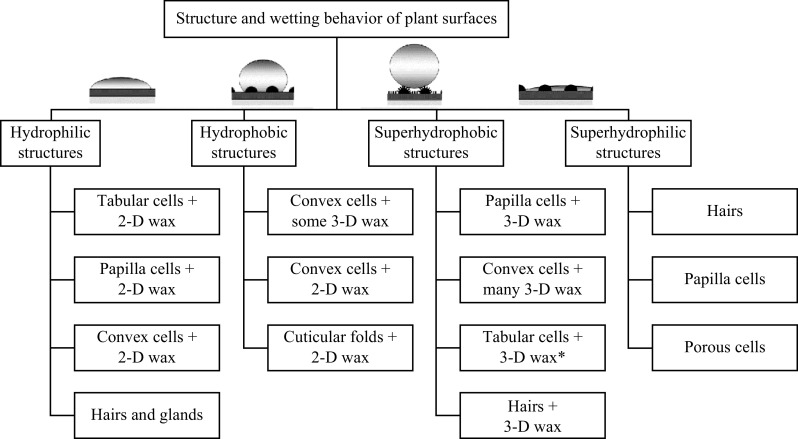
Four groups of plant surfaces wettability and the possible surface structures and structure combinations. The drawings used for the four groups are correlated to specific contact angles. Both the hydrophobic and superhydrophobic surfaces can be built by convex cells with three-dimensional waxes on it, but only a dense layer of wax crystals results in superhydrophobicity

The wettability of plant surfaces plays a crucial role in their interaction with the environment. A physical constraint of water plants is that they must be hydrophilic, in land plants, the whole range from superhydrophilic to superhydrophobic occurs (Fig. 20).

Hydrophilic and superhydrophilic surfaces (contact angle—if measurable—between 0° and <90°) are known from all aquatic plants and many surfaces of land plants which usually have a papillate cell morphology and cuticular folds, but also from leaves with flat, tabular cells. Surfaces covered by wax may become hydrophilic with the erosion of theses layers (e.g., *Carnegiea,* Fig. 1a), a very common process in many plants (see below). Modified wax layers may even become superhydrophilic like in the Atacama desert cactus *Copiapoa cinerea* adapted to fog harvesting. Hydrophilic behavior in flower petals of the daisy family (Asteraceae) and their polymer replica was analyzed in detail by Koch et al. [124] and provides information for biomimetic applications (see Sect. 7).

**Fig. 21:**
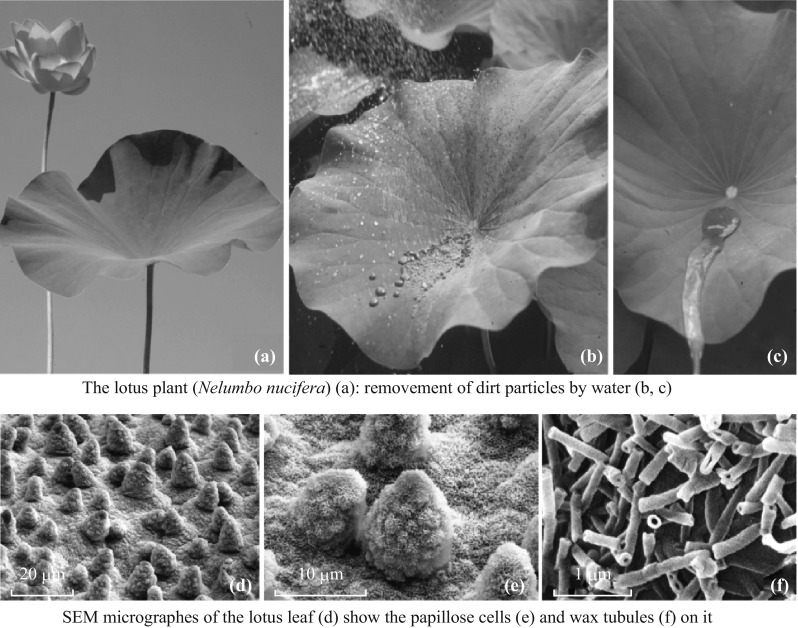
Superhydrophobic and self-cleaning surface of Lotus (*Nelumbo nucifera*). A flowering plant of Lotus (**a**), a lotus leaf contaminated with clay (**b**) and removal of the adhering particles by water (**c**). The SEM micrographs (**d**–**f**) show the lotus leaf surface in different magnifications: (**d**) The papilla epidermis, (**e**) single cell papilla, and (**f**) the epicuticular nonacosan-10-ol tubules on the cell surface

Superhydrophobicity was discovered in terrestrial plants, and is one of the main and most obvious characteristics of most vascular non-aquatic plants [1]. Superhydrophobic surfaces cover hundreds of millions of square kilometers on our planet’s surface (see Sect. 1). One precondition is always a hydrophobic chemistry of the surface and hierarchical sculpturing on two to several levels [1, 2, 7, 19]—these are the only two essentials to lead to superhydrophobicity in organisms. In plants, it is generated—with only a few exceptions—by 3D wax crystalloids of the morphological and chemical diversity previously described and illustrated (Fig. 7). Superhydrophobicity in these cases is caused merely by a sculpturing on the second level. For reasons of stability under dynamic conditions and to minimize mechanical damage, an additional third hierarchical level (usually convex cell surfaces) is essential—the best example are the Lotus leaves (Fig. 21) with contact angles >160° and a tilting angle of less than 4° [15] (survey of recent literature in Ref. [1]). The various functions of superhydrophobicity are discussed in Sect. 6. However, there is a phenomenon that “hairy” surfaces with cellular trichomes only covered by non-structured molecular films also commonly cause this effect. The classical examples are the superhydrophobic leaves of the Lady´s Mantle (*Alchemilla*) analyzed in detail by Otten and Herminghaus [125]. This is so obvious and often observed, that the names “Lady´s Mantle” as well as “Alchemilla” refer to the superhydrophobic properties. The “Alchemilla-type” of superhydrophobicity also occurs in the feathers of many birds.

**Fig. 22:**
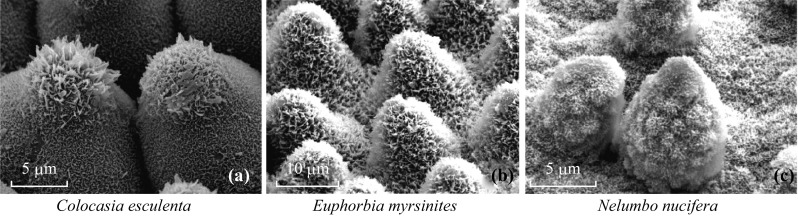
Extremely superhydrophobic leaf surfaces of (**a**) *Colocasia esculenta*, (Contact angle 164°) *Euphorbia myrsinites* (Contact angle 162°), and Lotus *Nelumbo nucifera* (Contact angle 162°), data from [7]. Leaves of all three species are characterized by convex (**a**, **b**) to papillose (**c**) cells, covered by three-dimensional wax crystals

A list of extremely superhydrophobic plant surfaces was provided by Neinhuis and Barthlott [7]. Amongst the extremes—all measured with the same equipment under the same conditions—are plants from most different relationships. Examples are Indian Cress (160° *Tropaeolum*), grasses (161° *Elymus*), Ginkgo trees (161° *Gingko*), Lotus (162° *Nelumbo*, Fig. 22c), Californian Poppy (162° *Eschscholtzia*), Aroids (164° *Colocasia*, Fig. 22a), and *Euphorbia myrsinites* (162°, Fig. 22b). Some monocotyledons of the family *Alstroemeriaceae* (“Peruvian Lilies”) exhibit extremely high contact angles caused by parallelly oriented wax platelets; the extreme was measured in a climbing *Bomarea* reaching a static contact angle of 169° [126].

Superhydrophobicity is usually not very persistent in plant surfaces. Leaves, with a limited life span, do usually not last longer than one year. Persistent leathery leaves (like in many Mediterranean and tropical climates) are usually hydrophilic—but they may start to be hydrophobic in their earliest developmental stages. This dynamics can be measured in the persistent leaves of *Welwitschia mirabilis*, a single leaf growing over two centuries from its base. They start being superhydrophobic exhibiting tubular nonacosan-crystals which eroded after the first year and the leaf becomes wettable [1]. Plums are covered by a mechanically delicate whitish wax cover—touching it with a finger is enough to destroy the structure and thus the “glaucous” color. An extreme provides the chemically complex brilliant white waxy coating of the succulent desert plants of the genus *Dudleya* (e.g., *D. brittonii*): it only takes a rain drop to destroy the instable coating [66]. *Dudleya* is not self-healing, in contrast to other plants, where a wax cover destroyed may regenerate within hours.

**Fig. 23:**
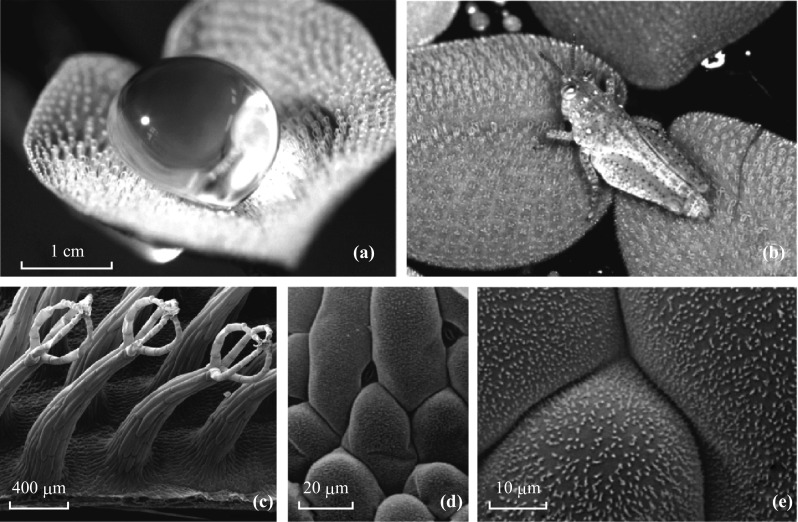
Air-retaining surfaces of the floating fern *Salvinia.* **a** A water droplet on the upper leaf side of *Salvinia biloba* and **b** a mimicking grasshopper *Paulinia acuminata* feeding on the leaf, even the grasshopper has a wax surface and is superhydrophobic. The SEM micrographs (**c**–**e**) of the leaf surface of *Salvinia* show the multicellular hairs **c**, and in higher magnification (**d**) the epidermis cells, and the wax rodlets (**e**) on the epidermis cells

Water plants are superhydrophilic, but when they rise above the water level they exhibit their old evolutionary potential, e.g., the flowering stalks of the watermilfoil *Myriophyllum* become superhydrophobic like the unique flowers of *Nymphoides* [1]. Floating species like *Salvinia* or *Pistia* are usually superhydrophobic. A striking example is illustrated in Fig. 23b: the grasshopper *Paulinia acuminate* feeds exclusively on *Salvinia* and optical mimics its color and surface (camouflage)—but it also is superhydrophobic based on wax crystals, the same adaption to its semiaquatic habitat as its host plant [1, 127].

Only few surfaces like lotus leaves have a very stable superhydrophobicity. As indicated in the introduction, superhydrophobicity usually disappears in aging biological surfaces and they become wettable (compare, Fig. 1a).

## Functional Diversity of Plant Surfaces

**Fig. 24:**
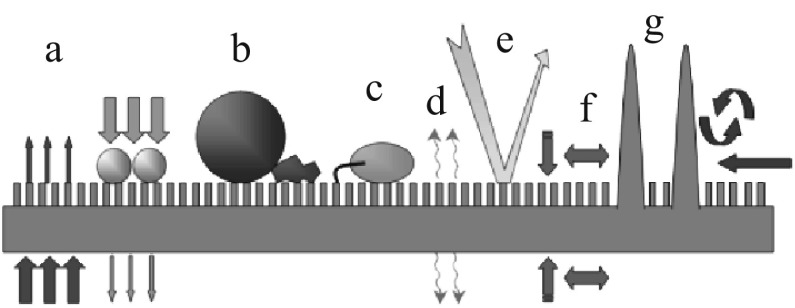
Schematic of the most important functions of the plant boundary layer on a hydrophobic micro-structured surface: **a** Transport barrier: limitation of uncontrolled water loss/leaching from interior and foliar uptake, **b** surface wettability, **c** anti-adhesive, self-cleaning properties (lotus effect): reduction of contamination, pathogen attack, and reduction of attachment/locomotion of insects, **d** signaling: cues for host-pathogens/insect recognition and epidermal cell development, **e** change of optical properties, **f** mechanical properties: resistance against mechanical stress and maintenance of physiological integrity, **g** reduction of surface temperature by increasing turbulent air flow over the boundary air layer (modified after Ref. [75])

The surface of plants is the critical interface for the interaction with the environment and fulfills many and most different functions (Fig. 24). Aspects and literature have been summarized by [1, 70, 71, 76, 128–130]. The surfaces are usually multifunctional and each attempt to categorize them is thus unrewarding [83, 131]. We simplify this in the following seven categories.

### Mechanical Properties

The cuticle itself is a highly sophisticated chemically stable layer which serves as an elastic mechanical protective structure [74, 128]. The shape of a ripe tomato is maintained to a high degree by its thin cuticle. Hierarchical surface structures fulfill various mechanical tasks; they can reduce the ability of insects to walk (e.g., fish-hooked hairs on bean leaves against aphids, trapping surfaces in insectivorous plants or flowers of *Arum*) or increase the ability of insects to walk, as is the case in flowers and even orient the visitors with cuticular folds [132]. Mechanochemical defense trichomes are common like in nettles (Urtica) or in Loasaceae (Fig. 14d).

### Attachment

Attachment mechanisms are most common in zoochorous fruits like burrs; complicated hooked spines, and hairs are evolved in many groups (Asteraceae, Krameriaceae, Pedaliaceae) [133]. A particularly refined attachment mechanism with extractable cellulose threads occurs in the seeds of the orchid *Chiloschista* [89]. Attachment plays a role in the interaction between pollen and its disperser and the stigma, where it becomes deposed. Climbing plants like Ivy (*Hedera*) or some highly specialized water plants that are growing on rocks in currents (e.g., *Podostemaceae*) exhibit attachment devices by glue-like adhesives, which are not really surface phenomena.

### Reflection, Absorption, and Transmission of Spectral Radiation

Reflection, absorption, and transmission of spectral radiation are of crucial importance for light harvesting and temperature control under insolation; colors as well as light reflection play an important role in the interaction between flowers and their pollinators.

#### Light Management

Absorption of light for photosynthesis is the precondition for plant life. Leaves need to collect electromagnetic radiation through photosynthesis while flowers need to harvest radiation in order to intensify their coloration to be more attractive for pollinators. In these processes, the architecture of the plant surfaces plays an important role. While self-cleaning surfaces often combine convex- or concave-shaped epidermal cells with water-repellent 3D waxes, light-harvesting leaf surfaces often possess only convexly shaped epidermal cells. Specifically plants under low-light conditions reduce the loss of light due to specular surface reflection by increasing the transmittance of energy via multiple reflections between the surface structures [134]. An extreme example is the “luminescent moss” *Schistostega* living in caves with batteries of spherical cells collecting the faintest light-like lenses [135]. However, convexly shaped cells combined with a cuticular folding on top are well known for petal surfaces, especially in angiosperms [2]. These cuticular folds were thought to reduce the surface reflection [136], act as a specific optical signal for pollinators [2] and cause iridescence generated through diffraction gratings [137].

#### Coloration Signals

**Fig. 25:**
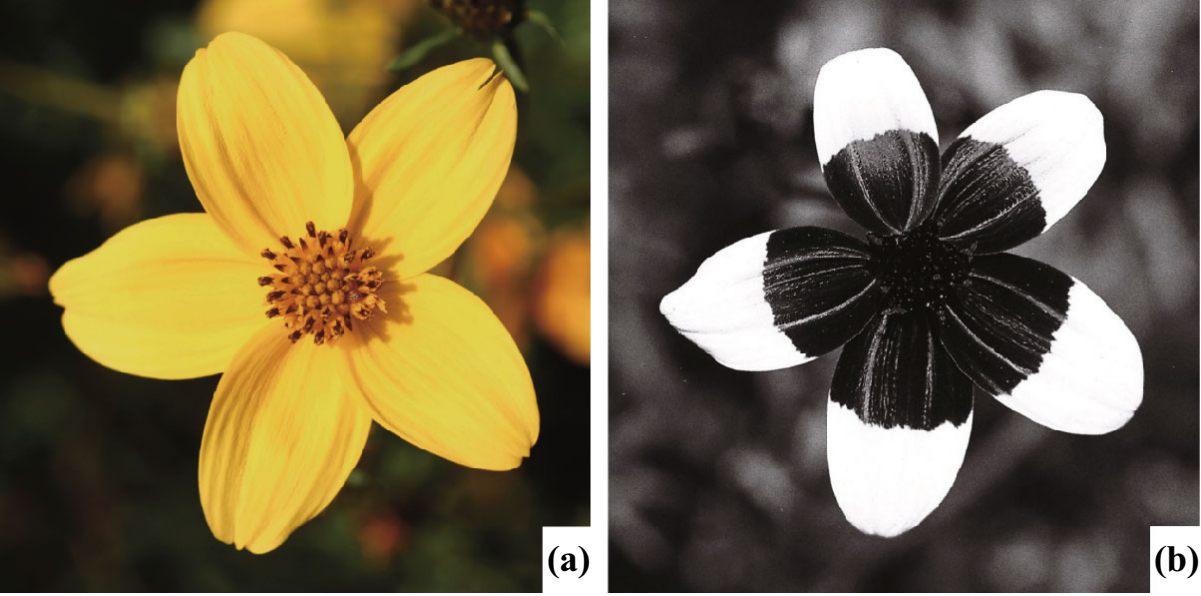
Optical properties. **a** Flower of *Bidens ferulifolia* in the visible light for human eyes. **b** An ultraviolet image of the same flower is shown. Reflection and absorption of radiation are primarily influenced by chemical composition, but surprisingly also by the hierarchical surface architecture

The intriguing interaction between flowers and their pollinators is the reason for the evolution of most refined coloration signals (e.g., Lloyd and Barett [138]). The spectrum comprised virtually all grades of reflections (from black to white) and all visible colors, even metallic-mirror like surfaces have been evolved in the orchid *Ophrys speculum*, thin layer iridescences like in our example (Fig. 4a). To intensify their color signal, some flowers use refined surface structures. The surface architecture of the flowers of the so called “Johnny-jump-up” *Viola tricolor* is an excellent model for this color intensification process. *Viola* possess extreme papillated epidermal cells (aspect ratio about 2.93) covered with a fine cuticular folding (width about 0.26 µm). This surface topography acts as a light-trap for incident light by reducing the specular reflection on the surface. Via this surface design more light passes through the cell wall. Furthermore, the extremely steep cell walls (papilla tip angle about 26°) cause an optimized scattering of the light within the petal (scattering angle of about 170°). This allows the absorption of more light by the pigments, which further results in the intensification of the color signal. Such a surface is highly interesting for biomimetic applications, for example, in solar panel development. Flowers also use optical signals in the ultraviolet range, not visible to the human eye, but visible to visiting bees: *Bidens ferulifolia* in Fig. 25 provides an impressive example. Large databases on ultraviolet reflection of flowers are published [139–141]. Usually it is assumed that the reflection is determined only by chemical compounds (flavonoids) within the living cells (vacuoles), but we have recently shown that UV-patterns are also supported by the surface structure. Another phenomenon caused by surface structures is iridescence of some leaves like in the fern *Elaphoglossum* (Fig. 4a). This phenomenon seems to occur mostly under low-light intensities of tropical rain forest understories, but is also known in some flowering plants, e.g., the petals of some tulips [137, 142]. Camouflage (like in the grasshopper *Paulinia* in Fig. 23b) plays an important role in desert plants (mainly South African Aizoaceae like the “living stones” *Lithops*): not only characteristics such as color, but also the microstructure of the surrounding environment is mimicked with high precision by their surfaces.

#### Temperature Control Under Insolation

The plant surface can only tolerate temperatures of up to 45 °C for living tissue. As for example, a car top heats up to more the 60 °C on a hot summer day, avoiding such temperatures is a crucial challenge for living tissue under solar radiation. Transpiration cooling is an obvious adaption, but inevitably connected with loss of water. Obviously, the highly reflective wax coatings or an indumentum of hairs decreases the heating [143–146]. An important aspect of temperature control—apart from reflections—may be the increase of turbulences by mechanical turbulence aids (e.g., the diversity of leaf margins) under dynamic (wind) conditions, which enhance the temperature exchange between the cooler air and the heated isolated leaf surfaces [147–150]. It is not by chance that succulents of the arid hot regions exhibit a high complexity of hierarchical surfaces sculpturing.

### Reduction of Water Loss

Many of the land plant surface structures must be observed in context of reduction of water loss—a well-researched field. The cuticle, wax layers, and an indumentum of dense hairs play the crucial role in preventing loss of water, in some desert plants like *Sarcocaulon* the waxy layer might be more than one centimeter in thickness. This function is rather irrelevant for biomimetic applications: non-organic materials like metals and synthetic polymers provide better solutions. *Uptake of water* is connected with superhydrophilicity (see Sect. 6.5).

### Superhydrophilicity

**Fig. 26:**
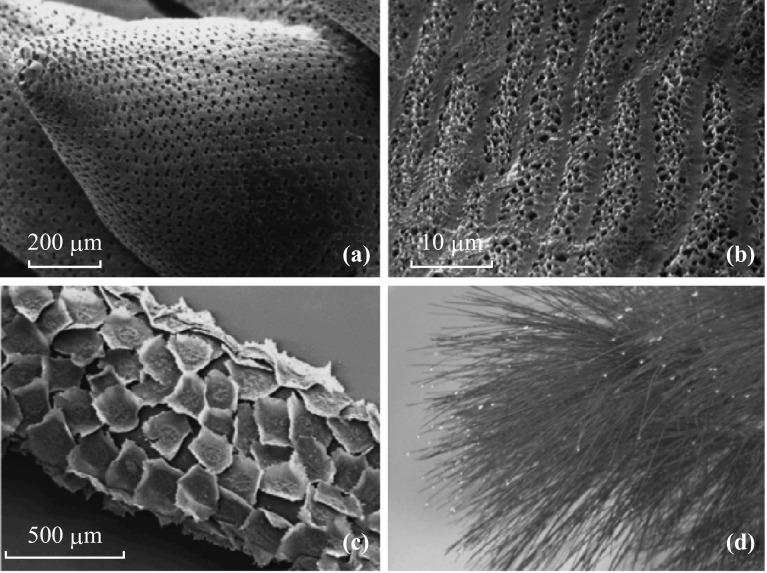
**a** The water absorbing pores of a *Sphagnum squarrosum* moss. **b** The water adsorbing porous surface of the moss *Rhacocarpus purpurascens*. **c** The epiphytic growing Spanish moss (*Tillandsia usneoides*). **d** Water droplets in the needles of the Canary Pine (*Pinus canariensis*) harvesting fog out of the trade wind clouds

Superhydrophilicity is a physical constraint in water plants, in land plants it is mainly connected with the uptake of water (roots, epiphytic, and fog-collecting plants). Mosses without conductive tissue are superhydrophilic for an “ectohydric” water uptake, the most refined structures are found in *Rhacocarpaceae* (Fig. 26a, b) [151, 152]. All epiphytes (plants growing on other plants without contact to the soil) depend on rain, fog, and dew and they are all hydrophilic and mostly even superhydrophilic [1]. The best known examples are the hair-covered leaves of bromeliads (Fig. 26c). But there are many more plants with fog and dew collecting abilities in certain semiarid regions like the Atacama and Namib desert [153–155] which are of particular interest for biomimetic application (fog collectors) (Fig. 26d). A remarkable case is the leaves of understory plants in tropical rain forests, like the superhydrophilic *Ruellia devosiana* with a strong convex sculpturing of its epidermis cells. Glands on the leaf surface release saponins, causing a thin hydrophilic coating, supporting a rapid spreading of water droplets [10].

Some superhydrophilic water plants have optimized surfaces and morphologies to resist water flows. By exhibiting grid-like leaves the Madagascar Laceleaf (Fig. 5c) reduces its flow resistance. The superhydrophilic wave-swept giant seaweeds (Phaeophyta) are adapted by their morphology to reduce drag [156]. Their leathery thallus is covered by a mucilage; recent work indicates that the superhydrophilicity of the mucilaginous surface acts as a drag-reducing agent like the mucilage of fish.

### Superhydrophobicity

**Fig. 27:**
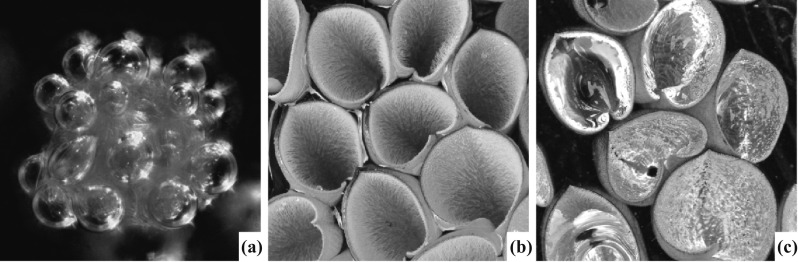
Compartmentation of surfaces generating small functional units for air retention: (**a**) in microscopic dimension in the seed of *Aeginetia indica* illustrated in Fig. 3 for temporary floating. A particularly refined compartmentation system (**b**, **c**) exhibits *Salvinia cucullata* in a macroscopic dimension: the superhydrophobic leaves (diameter ca. 1.5 cm) form hood-like compartments (**b**), submersed in water, (**c**) each leaf holds a very stable large air bubble

Superhydrophobicity is one of the most obvious characteristics of many land plant surfaces and may serve different functions. In particular, the structures for air retention under water are highly complex (e.g., *Salvinia*, Fig. 23); and they are often combined with a superimposed compartmentation [1] of the whole organ surfaces from the microscopic to the macroscopic level (Fig. 27).

**Fig. 28:**
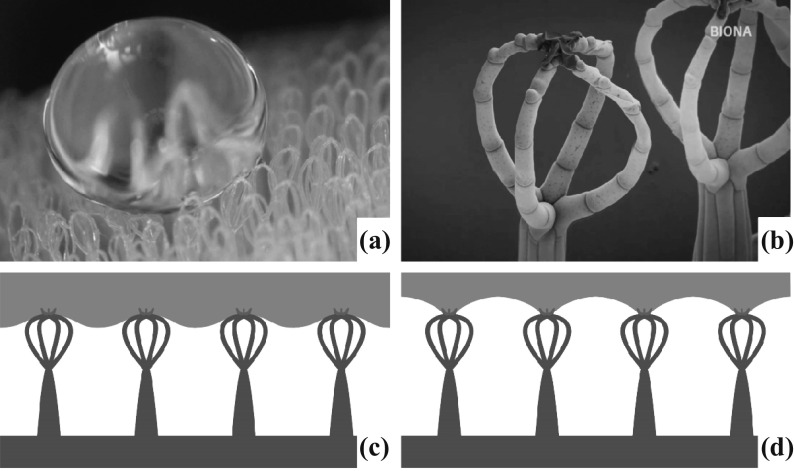
The Salvinia paradox of chemical heterogeneities. **a** Water droplet on the eggbeater-shaped trichomes of *Salvinia molesta*. The deformations at the contact points of the droplet with the tips of the trichomes indicate an adhesion of the water, the droplet does not roll-off an inclined surface. **b** Colored SEM-image of the wax-covered superhydrophobic trichomes (*green coloration*) and the terminal hydrophilic four anchor cells for pinning the air–water interface. **c**, **d** Schematic of the air–water interface under dynamic hydrostatic conditions. **b** is from Barthlott, Bertling, Schoppa, Vogt 2011. (Color figure online)

(i) Unwettability per se to avoid wetting for mechanical reasons: thin laminar leaves become too heavy when wetted. This is probably an important determination leading to the adaptation of superhydrophobicity in larger leaves in rain forests (e.g., *Cibotium* [1])—an extreme provides the Titan Arum *Amorphophallus* (Fig. 1b).
(ii) Reduction of adhesion for insects—usually connected with superhydrophobicity—is caused by epicuticular wax crystals in insect-catching plants (e.g., *Sarracenia, Nepenthes*) or flowers which trap their pollinators (*Ceropegia, Aristolochia*) or to avoid unwanted nectar thieves (*Fritillaria, Lapageria*). Superhydrophobicity itself is not the primary function in these surfaces.
(iii) Reduction of adhesion to avoid contamination (lotus effect, shown in Fig. 21) enables the plants to be cleaned from any kind of contaminating particle by raindrops, it is probably the most important function of superhydrophobicity in plants [1, 15, 157, 158]. Biologically, such surfaces in plants and animals should be primarily seen as a defense mechanism against fungal spores and the colonization with other micro-organisms.
(iv) Air layers for buoyancy. Non-persistent air layers for temporary buoyancy in water play an often underestimated role in very small seeds (see Fig. 27a) and spores which may fall or are dispersed into water.
(v) Air layers for gas exchange (respiration) play mainly a role in animals (survey in Refs. [159, 160]), and also in plants like *Salvinia* which become temporarily inundated under water.
(vi) Air layers for fluid drag reduction. We could show (survey in Ref. [1]) that persistent air layers reduce the friction. The mechanism of this drag reduction is fairly simple: the air layer serves as a slip agent. On solid surfaces, the velocity of the water directly at the surface is zero due to the friction between the water and the surface molecules. If an air layer is mounted between the water and the solid surface, then the water streams over the air layer. The viscosity of air compared to water is 55 times lower, because of this the air layer serves as a slip agent and the drag is reduced. Air layers as slip agents have evolved in many insects and play probably no role in plants. However, *Salvinia* provides the best example for the maintenance of permanent air layers under water (salvinia effect), showing the four essential criteria for air retention: hydrophobic chemistry, hair-like structures, undercuts and elasticity of the structures. Also the fifth, non-essential criteria, hydrophilic chemical heterogeneities of anchor cells within its superhydrophobic surface (Salvinia paradox, Fig. 28) could be found in some *Salvinia* species [67, 161–168].

### Anti-adhesive “Slippery” Surfaces and Aquaplaning

Many plants have evolved special structured surfaces which hinder the attachment of animals, especially insects, to protect themselves against herbivores [169]. Most insects possess two different types of attachment structures, claws and adhesive pads [170, 171]. Whereas the former are used to cling to rough surfaces, the latter enable them to stick to perfectly smooth substrates. One strategy to reduce the attachment of insects is the secretion of epicuticular waxes which assemble into three-dimensional micro-structures. The other strategy is the development of a slippery surface by inducing aquaplaning.

**Fig. 29:**
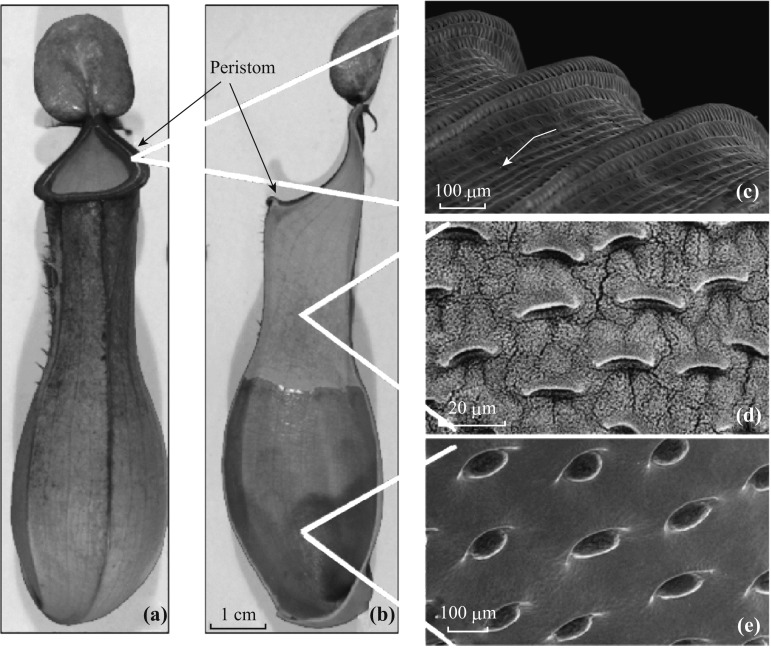
Pitcher traps of the carnivorous plant *Nepenthes alata*. In (**a**) the complete pitchers trap of *N. alata* and in (**b**) a longitudinal cut through the trap are shown. **c** Parallel ridges of the hydrophilic peristome. The *arrow* indicates the direction toward the inside of the pitcher. **d** The waxy and slippery surface inside the trap with inactive stomata. **e** Glands located in the digestive zone at the *lower part* of the trap are shown. **c** was kindly provided by Holger Bohn

“*Aquaplaning*” Several *Nepenthes* species do not possess a waxy layer, but are nevertheless fully functional insect traps. It was found that *Nepenthes* evolved another capture mechanism which is based on special surface properties of the pitcher rim (peristome) (Fig. 29a, b). The peristome is characterized by a regular microstructure with radial ridges of smooth overlapping epidermal cells, which form a series of steps towards the pitcher inside. The peristome ridges (Fig. 29c) mostly extend into tooth-like structures at the inner edge, in between which large glands (extrafloral nectaries) are situated. Secretion of nectar by the glands attracts small insects, and also leads to a hydrophilic coverage of the surface. The plant surface microstructure combined with hydrophilic surface chemistry renders the pitcher rim completely wettable. Water droplets spread rapidly and form homogeneous thin films, which make the peristome extremely slippery for insects. When the peristome is wet, the fluid films prevent the insects’ tarsal adhesive pads from making close contact with the surface, similar to the aquaplaning of a car tire on a wet road. In addition, the anisotropic microstructure of the peristome surface allows interlocking of claws only while the insect is running towards the pitcher inside, but not on the way out [172]. Under natural conditions the slippery water films are caused by rain, condensation, and nectar secretion. In contrast to this, dry peristomes are not slippery for insects. This weather-dependent variation of peristome slipperiness leads to an intermittent and unpredictable activation of *Nepenthes* pitcher traps, which might make the evolution of specific avoidance behaviors more difficult [173].

## Biomimetic Application

Bionics or biomimetics describes the processes in which structures and concepts evolved by living organisms are taken and implemented into technologies (surveys in [1, 11, 19]). Bionics or biomimetics is an old field (historical survey in Ref. [11]), but functional surfaces came surprisingly very late to bionic applications. The first example was the hook-and-loop fastener by the Swiss engineer Georges de Mestral in the 1950s, popularly known as Velcro^®^. It is based on plant surfaces (burrs). The drag-reducing riblets of the shark skin were analyzed in the 1980s [17] and together with the self-cleaning lotus-surfaces [2, 15] ushered in a new era of biomimetic applications—including the swimming competition in Olympic games (see Sect. 7.6).

“*Surfaces*” in engineering are thin boundary layers, and they are exposed to the harsh physicochemical and mechanical influences of the environment. Thin layers, and in particular, the biomimetic interesting hierarchical structures, have a restricted stability and durability. Nanocoatings play a most important and increasing economic role (compare Ref. [174]). We live—in the literal sense of Mark Twain’s novel—in “the gilded age” of surface applications. The bulk of a technical solid is usually stable, but every homemaker knows the difference between the durability of a silver spoon in contrast to a silvered utensil. A medieval iron pan could still be used in the kitchen over the next centuries—but a polymer-coated anti-adhesive pan lasts a few years. The extremely long lifespan of spectacle glasses is today limited by the sophisticated and convenient anti-reflex coatings. In colloquial language, there is an obvious difference between “solid” and “superficial.” We need coating technologies—but durability and persistence are major technical challenges for engineers and material scientists in a world of decreasing natural resources which must be used sustainably.

Section 7 is organized along using the same scheme as in Sect. 6 on plant functionality.

### Mechanical Properties

Mechanical Properties play a very important role in biomimetic composite materials—but are nearly negligible for surfaces. Technical materials available today (polymers, metals, etc.) have often superior mechanical properties.

### Attachment

Attachment mechanisms of plants were the first biomimetic surface application and have been around since 1958 (see above) with the Velcro^®^ hook-and-loop fasteners. Plants, in contrast to animals (e.g., geckos) are only a limited source of inspirations for attachment mechanisms.

### Reflection, Transmission, and Absorption of Spectral Radiation

Reflection, transmission, and absorption of spectral radiation are of great importance for biomimetics. The applications concentrate on light harvesting by bioinspired surfaces of solar panels. An appropriate model for such surfaces is the petals of *Viola tricolor* and it is related species (see Sect. 6.3). They show a highly reduced reflection of light, an optimized scattering within the petals; additionally, they are superhydrophobic, showing a contact angle of 169° [175]. Temperature control under insolation (e.g., car tops, roofs) is another field of interest. Coloration technologies have always been bioinspired (like indigo and purple as ancient dyes used for millennia). Flowers exhibit sophisticated structures to intensify colors by hierarchical structuring. However, most inspirations are provided by animal coloration, like the iridescence of peacock feathers or butterfly (*Morpho*) wings. Butterflies also provide ultrablack colorations [176] like the Gaboon viper [177]. It is not by chance that the darkest technical black (Vantablack) is produced by carbon nanotubes.

### Reduction of Water Loss

Reduction of water loss is essential for terrestrial plants—but like the many mechanical properties (see Sect. 7.1) of minor importance for biomimetic applications. Technical materials like an aluminum foil prevent loss better than a cuticle.

### Superhydrophilicity

Superhydrophilicity has recently become relevant for technical applications, which are most diverse. Biomimetic fog-collecting meshes and other devices will probably play an increasing role in certain arid regions (e.g., Chile, Namibia), and it was shown that a biomimetic superhydrophilic hierarchical structuring increases the collection efficiency [154, 155, 178]. Koch et al. [124] have shown that the hydrophilic *Gazania* petal structures provide a model for the design of microfluidic devices for small volume liquid transport by capillary forces; beneficial in both fog harvesting and microfluidic devices.

Evidently, superhydrophilic surfaces are wettable, but the water film evaporates very fast and leads to the fast drying “no-drop” glasses (e.g., Alltop^®^) and a sophisticated façade paint (StoColor Dryonic^®^). Drag reduction by mucilaginous surfaces seen in fish also plays a technical role; we have seen evidence in seaweed surfaces that plants have also evolved a similar technology.

**Fig. 30:**
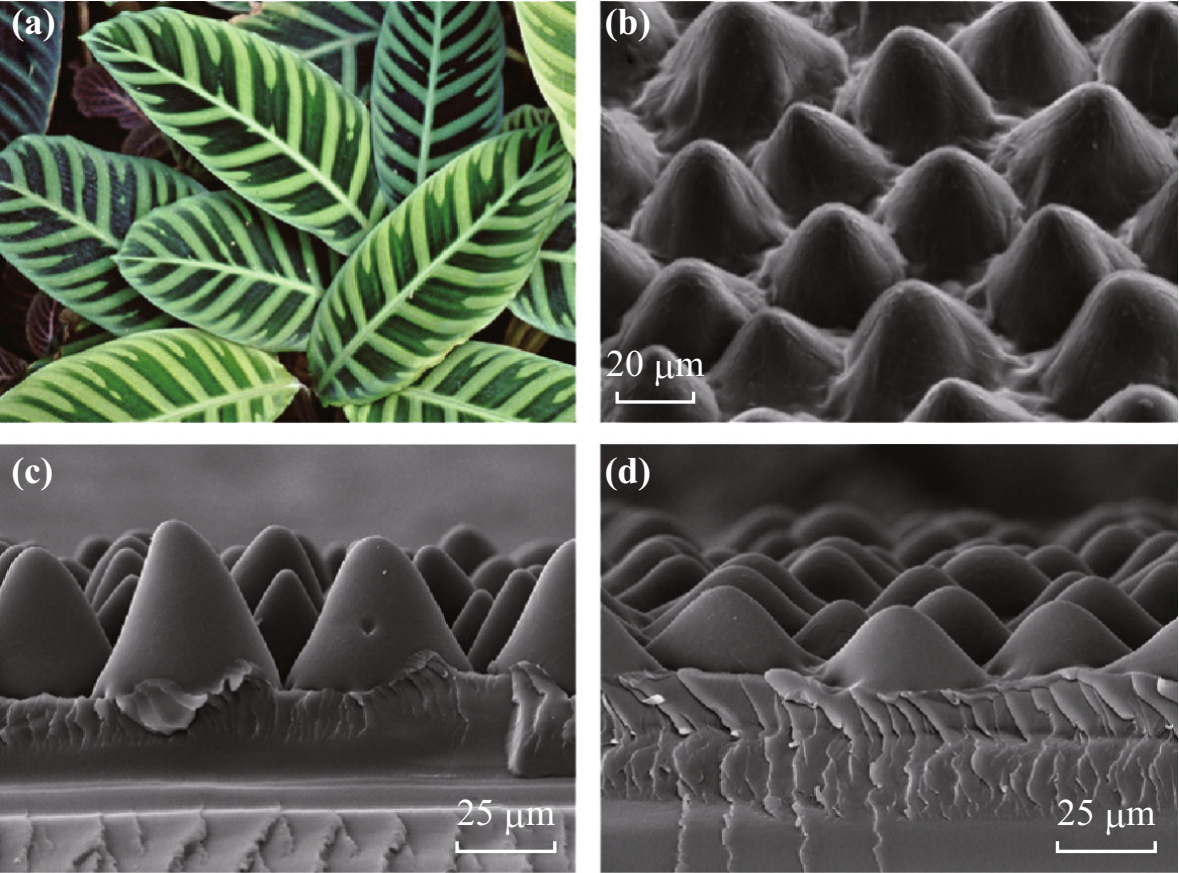
**a** *Calathea zebrina* leaves with their *light* and *dark green* pattern. **b** SEM micrograph of the leaf surface (tilt angle 45°). SEM of the replicated dark leaf areas (**c**) and bright leaf areas (**d**) in side view (90° tilt angle). The visible *color* differences in the leaf are mirrored in the different *shapes* of the cell papillae of the *light* and *dark* surfaces areas. (Color figure online)

The form and dynamics of bouncing droplets [93, 179] are influenced by surface chemistry and structure: superhydrophilic surfaces may prevent splashing, an important effect for biomedical applications (hygiene) or agriculture [180]. In plants, this phenomenon was analyzed in Calathea zebrina, a hydrophilic tropical understory plant, and its corresponding polymer replica (Fig. 30) by [122]. The anti-splashing effect is promising for biomimetic applications.

### Superhydrophobicity

Superhydrophobicity, the most obvious feature of many plant surfaces, has an increasing and most diverse role in biomimetics as early as the 1990s (survey in Ref. [1]). Superhydrophobicity does not exist in abiotic nature and is one of the most important biomimetic applications under different aspects. Superhydrophobicity is generated in many plants by nonacosan-10-ol tubules with a diameter of 110 nm (Fig. 13). It could be shown that carbon nanotubes can also be used to generate superhydrophobic, even air-retaining surfaces.

- Pure water repellency (umbrellas to textile building) is usually applied by sprays providing a sculpturing by nanoparticles on, e.g., textiles (which are intrinsically hierarchically structured). It is obvious, that dry conditions further reduce corrosion, and anti-corrosion coatings are the focus of technical applications [181]. This also applies for anti-icing properties. A whole range of products and techniques are available, mostly coatings or sprays, the product names often indicate their function (e.g., ‘NeverWet’). Dry surfaces may allow new dimensions in textile-based architecture owing to weight reduction during rainfall. Anti-ice and anti-frost performance is increasing in importance [182, 183].
- Self-cleaning Lotus Effect^®^ surfaces are farout most important [1] and a vast literature exists since 1997 (see Ref. [19, 52, 184–186]). It is also one of the most important applications in façade paints, with the added advantage that they remain functional for several decades.

The lotus effect is evolved in plants predominantly as a defense mechanism against pathogens: the attachment, e.g., of fungal spores is dramatically reduced [187], and no water films are available as a precondition for a colonization or the formation of biofilms. This has an unexpected application: in commercial agriculture all plants treated with pesticides by spray application surfactants have to be added. It was shown that the application of the solution containing only the surfactant (without the active ingredient) increases the chances of infection significant [187–192]. This phenomenon will hopefully lead to the development of a new generation of less aggressive, bioinspired surfactants for pesticide application.

- Superhydrophobic plants surfaces, like those of *Salvinia*, have a striking capacity to collect and adsorb spilled oil from water [193], a basis for biomimetic materials. Hydrophobic sand coating can be used to control deep drainage in tailings [194].
- Air-retaining Salvinia^*®*^ Effect surfaces are a recent development, only prototypes exist [1, 195, 196]. Fluid drag reduction of up to 30% in a hydrodynamic water channel has been measured [197]. The main application is seen in ship hulls: container ships transport about 80% of the global goods, and a reduction of the fuel (oil) consumption of 125 million tons and of 395 million tons of CO_2_ is estimated. A novel technology using air-retaining grids has recently been introduced and can be combined with the existing-refined micro-bubble technologies: micro-bubbles adhere to the grid-surface and the air layer under the grid can be regenerated. It is obvious that a permanent intact air layer prevents biofouling. Oil pollution is an unfortunate and unavoidable problem in our oceans. One possible solution is to create a hydrophobic barrier at the waterline of the ship to separate the ship hull from the water interface to avoid oil from creeping into the structures and suppressing the air.

**Fig. 31:**
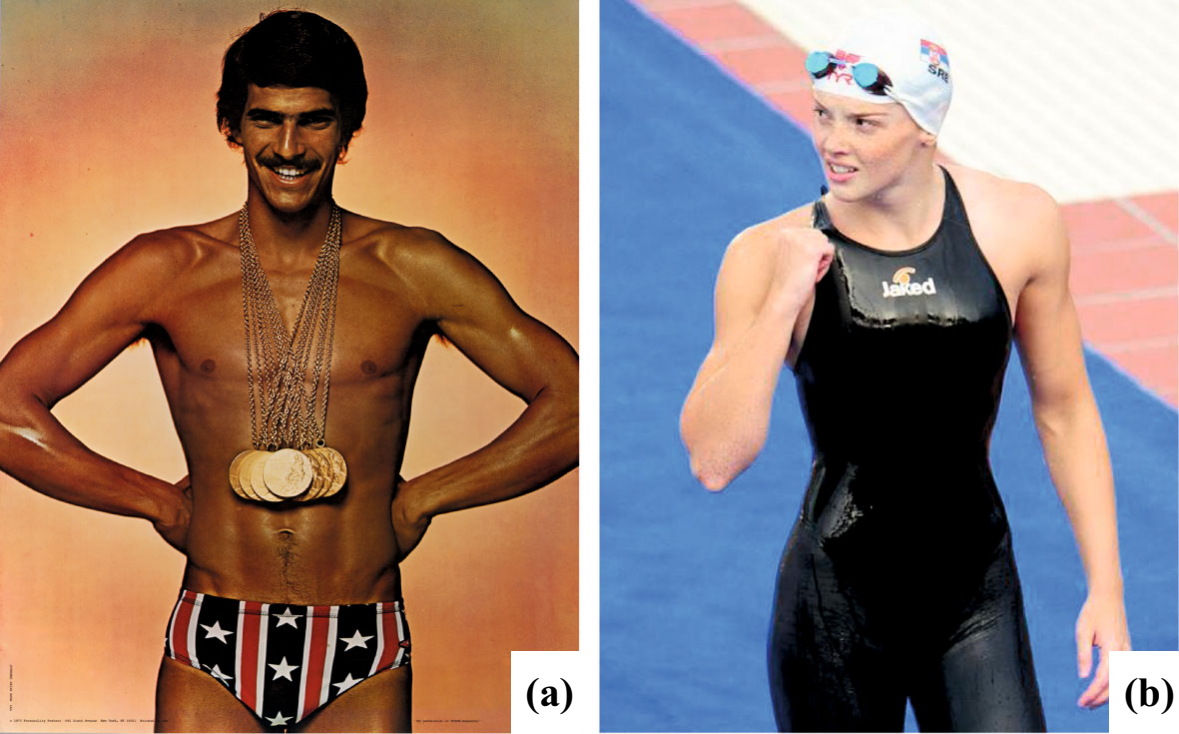
Some Olympic records today are also a triumph of bionics. Mark Spitz (**a**) wore his famous pre-bionic swimsuit in the 1972 Olympics in Munich. In contrast, biomimetic swimsuits covering almost the entire body were popular after 2000; the suits were based on the biomimetic drag-reducing shark riblet technology. At the World Swimming Championship in July 2009 in Rome almost all world records were broken using full-body biomimetic polyurethane swimsuits. Subsequently, the length of swimwear was limited by the World Swimming Federation FINA in December 2009. But almost full-body swimwear is back today, e.g., the swimming suit of Nađa Higl (**b**): somewhat smaller than in 2009, but still covering large parts of the swimmers. Today, it seems to be a high-tech combination of chemically heterogenic surfaces: shark riblets (drag reduction about 3%) and superhydrophobic salvinia effect (drag reduction about 30%) areas. For divers, drag reduction plays no role—the divers in Rio de Janeiro 2016 were still dressed like Mark Spitz in 1972. *Sources* **a** kindly provided by the National Portrait Gallery, Smithsonian Institution, **b** from Wikipedia Commons

Biomimetic drag-reducing boat surfaces based on shark skin riblets have been applied in America’s Cup contests in 1987 and 2010; the Speedo Fastskin^®^ swimming suits introduced in 2000 were adopted by almost all gold medal winners in the Olympic Games in Beijing 2008 (Fig. 31). But riblet structures reduce the friction only about 3%, the more complex Salvinia^®^ Effect technologies about 30%. Swimwear covering the body almost totally was banned in 2009; however, there is some evidence that in the Olympic Games in Rio 2016 a refined technology of bionic swimming suits combining superhydrophobic salvinia effect areas with shark riblet areas may have been applied. Underwater persistent air layers will probably fulfill many other functions like gas exchange (“plastron” in biology) or even refined sensory functions like in the backswimmer *Notonecta* [198].

### Other Applications

As indicated in the preceding chapter, there are additional functions. Like the “aquaplaning” effect of the surfaces of the carnivorous *Nepenthes* plants which produce water films and become slippery to trap insects [172, 199]. In particular slippery liquid-infused surfaces (SLIPS) are of focal interest (e.g., Ref. [200, 201]).

## Living Prototypes: Evolution of Plant Surfaces and Biodiversity

The often used phrase “inspired by nature” for biomimetics is misleading: a nuclear reactor is inspired by natural nuclear fission or the nuclear fusion in our sun. Thus a nuclear reactor is inspired by nature—but not bionic. Biomimetics is exclusively based on living organisms and the evolution of some 10 million living prototypes (survey in Ref. [1]). Superhydrophobic surfaces occur exclusively in living organisms, which evolved hierarchical structured superhydrophobic surfaces based on a very limited selection of molecules. There is no evidence for the occurrence of superhydrophobic surfaces in anorganic *nature* (man-made technical products are excluded). Surfaces are the crucial interface between an organism and its environment—3.5 billion years of mutation and selection have created a stunning diversity in an estimated 10 million different species of plants and animals (survey in Ref. [1]). The two basic phases with contrasting physical constraints are life in water and life outside of water.

### Water Plants

Non-vascular primary water plants evolved in water (originally the oceans) a couple of trillion years ago and have no true conductive tissue. The groups are phylogenetically very different non-related clades; they comprise microscopic unicellular algae up to seaweeds which might reach a height of more than 45 m in *Macrocystis*. The common feature is their wettability (superhydrophilicity) and usually the absence of hierarchical structuring: flat and mucilaginous surfaces are characteristic. In certain groups of unicellular algae (e.g., Coccolithophores and Diatoms) complex surface structures occur, but usually embedded in mucilaginous or plasmatic covers: they are probably not the environmental interface.

**Fig. 32:**
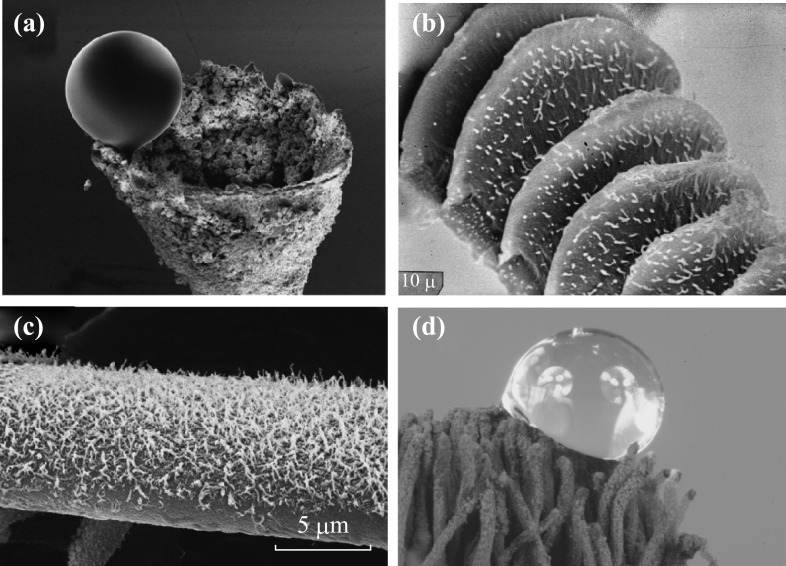
Superhydrophobicity in lichens mosses, fungi, and slime mold. **a** Fruiting body of the lichen *Cladonia chlorophaea* with water droplet. **b** Peristome of the moss *Funaria hygrometrica*, covered by wax rodlets. **c** The aerial hyphae of the *gray* mold (*Botrytis*) are covered by wax-like crystals. **d** The fruiting body (capillitium) of the slime mold *Stemonitis* possesses a superhydrophobic surface caused by a granular layer of unknown chemical composition

Vascular secondary water plants are plants of terrestrial origin, which recolonized aquatic habitats in fresh water (e.g., *Elodea, Potamogeton*) or even the oceans like seagrasses (e.g., *Posidonia, Zostera*). They exhibit the characteristic superhydrophilic flat surfaces, often becoming mucilaginous by biofilms, as primary water plants. However, they retain the ability of their ancestors to produce superhydrophobic surfaces when leaves or flowering shoots emerge from the water (e.g., in *Myriophyllum*). The ancestral non-vascular *Bryophyta* (mosses, hornworts, and liverworts) depend on the uptake of water through leaves and are superhydrophilic, occasionally exhibiting refined absorption structures like *Rhacocarpus* [152]. However, some (e.g., *Funaria hygrometrica*) have the ability to produce superhydrophobic wax covers in their spore capsules (Fig. 32b); the same is true for propagation structures of Lichens (Fig. 32a) [201] and the aerial hyphae of fungi (Fig. 32c) or even the capillitium of slime molds (Fig. 32d) [1].

### Land Plants

True land plants are the vascular or higher plants, a well-defined phylogenetic unit. They comprise the Ferns, Clubmosses and Horsetails, Gymnosperms (incl. Conifers), and the flowering plants (Angiosperms). Higher plants evolved with the conquest of land in the late Ordovician or Silurian, 430–500 million years ago and evolved mechanically stabilizing lignose vascular tissues and a protective polymer layer, the cuticle (see Sect. 3) on their stem, and leaf surfaces; as a consequence of this rather impermeability, surface pores (stomata) evolved to enable and control the gas exchange. It was shown by a phylogenetic analysis [1] that superhydrophobicity caused by epicuticular wax crystals evolved simultaneously with the conquest of land—a possibly overlooked evolutionary key invention for plant life outside of water and probably also for insects. One of the most common waxy substances is the fatty secondary alcohol nonacosan-10-ol, which is responsible for the superhydrophobicity of many ferns, conifers, or even lotus leaves as long ago as 250 million years [1].

Some 450,000 different species of plants are part of the astonishing biodiversity of our planet: including animals, some 10 million species—but we know of less the 20% of them. Plants have evolved most intriguing functional surfaces over millions of years, like in the Lotus (*Nelumbo*) or the floating ferns (*Salvinia*). Evolution is a slow process which has been spanning billions of years. Mutation and selection (“trial and error”) exploited all constructional possibilities within this time and with the limited materials. But million years of research and development for technical engineering today is not a possibility: the materials science has a goal of fabricating a particular product within a limited time, using experimental trial-and-error approaches, calculation, and modeling. We lose a high amount of biodiversity in our changing world and it has been brought to our attention [11, 203] that this also means the loss of biological role models, the “living prototypes” for engineers.

## Conclusions

The diversity of plant surface structures is a result of several billions of years of evolutionary processes. Plants evolved a stunningly high diversity of surfaces and functionality for their interaction with the environment—the self-cleaning properties of Lotus is only one example. Millions of years of mutation and selection, trial and error: free information for engineers and materials scientists.

Bionics is an old field of research and development starting around 1800—but surfaces played a surprisingly late role for biomimetic applications, the only exception is the hook-and-loop fasteners (“Velcro^®^”) in the 1950s based on burrs. The publication of the lotus effect in 1997 [15] created awareness by engineers and materials scientists, terms like “superhydrophobicity” came into use in the last two decades and opened a new era in surface technologies (survey in Ref. [1]). Surfaces play an increasing roll, the global market for nanocoatings is estimated to reach 14.2 billion US dollars by 2019 [173].

Biological surfaces have provided a remarkable number of innovations in the last three decades. Surface technologies have been largely influenced by research on biological interfaces and came rather late into focus of technical innovations. All data indicate we are only in the beginning of a new era of biologically inspired surface technologies.

Understanding biological surfaces is crucial—we have shown that we are still in the beginning of this process. But we are also in the beginning of a dramatic loss of biodiversity in the Anthropocene. Some 10 million different species (possible biological role models) exist—We lose a high amount of biodiversity in our changing world and it has been brought to our attention [11, 202] that this also means the loss of biological role models, the “living prototypes” for engineers. Bionics is another intrinsic value to the diversity of life which should be treasured.
